# A tailored approach to fusion transcript identification increases diagnosis of rare inherited disease

**DOI:** 10.1371/journal.pone.0223337

**Published:** 2019-10-02

**Authors:** Gavin R. Oliver, Xiaojia Tang, Laura E. Schultz-Rogers, Noemi Vidal-Folch, W. Garrett Jenkinson, Tanya L. Schwab, Krutika Gaonkar, Margot A. Cousin, Asha Nair, Shubham Basu, Pritha Chanana, Devin Oglesbee, Eric W. Klee

**Affiliations:** 1 Department of Health Sciences Research, Mayo Clinic, Rochester, Minnesota, United States of America; 2 Center for Individualized Medicine, Mayo Clinic, Rochester, Minnesota, United States of America; 3 Department of Laboratory Medicine and Pathology, Mayo Clinic, Rochester, Minnesota, United States of America; 4 Department of Biochemistry and Molecular Biology, Mayo Clinic, Rochester, Minnesota, United States of America; 5 Department of Medical Genetics, Mayo Clinic, Rochester, Minnesota, United States of America; 6 Department of Clinical Genomics, Mayo Clinic, Rochester, Minnesota, United States of America; German Cancer Research Center (DKFZ), GERMANY

## Abstract

**Background:**

RNA sequencing has been proposed as a means of increasing diagnostic rates in studies of undiagnosed rare inherited disease. Recent studies have reported diagnostic improvements in the range of 7.5–35% by profiling splicing, gene expression quantification and allele specific expression. To-date however, no study has systematically assessed the presence of gene-fusion transcripts in cases of germline disease. Fusion transcripts are routinely identified in cancer studies and are increasingly recognized as having diagnostic, prognostic or therapeutic relevance. Isolated reports exist of fusion transcripts being detected in cases of developmental and neurological phenotypes, and thus, systematic application of fusion detection to germline conditions may further increase diagnostic rates. However, current fusion detection methods are unsuited to the investigation of germline disease due to performance biases arising from their development using tumor, cell-line or *in-silico* data.

**Methods:**

We describe a tailored approach to fusion candidate identification and prioritization in a cohort of 47 undiagnosed, suspected inherited disease patients. We modify an existing fusion transcript detection algorithm by eliminating its cell line-derived filtering steps, and instead, prioritize candidates using a custom workflow that integrates genomic and transcriptomic sequence alignment, biological and technical annotations, customized categorization logic, and phenotypic prioritization.

**Results:**

We demonstrate that our approach to fusion transcript identification and prioritization detects genuine fusion events excluded by standard analyses and efficiently removes phenotypically unimportant candidates and false positive events, resulting in a reduced candidate list enriched for events with potential phenotypic relevance. We describe the successful genetic resolution of two previously undiagnosed disease cases through the detection of pathogenic fusion transcripts. Furthermore, we report the experimental validation of five additional cases of fusion transcripts with potential phenotypic relevance.

**Conclusions:**

The approach we describe can be implemented to enable the detection of phenotypically relevant fusion transcripts in studies of rare inherited disease. Fusion transcript detection has the potential to increase diagnostic rates in rare inherited disease and should be included in RNA-based analytical pipelines aimed at genetic diagnosis.

## Introduction

The uptake of next-generation sequencing for clinical testing has brought about a surge in the diagnosis of rare genetic disease. Approximately 18–40% of cases originally escaping a diagnosis with traditional genetic assays are now solved by exome-based DNA sequencing [1–3]. Despite such advances, a clear need remains for novel and improved methods that will further increase diagnostic rates and improve patient care. While whole-genome sequencing will likely lead to higher diagnostic rates, it remains less cost effective than exome sequencing and significant advances in understanding are required before its non-coding data can be harnessed for clinical practice [4].

Recently, RNA-Seq has been promoted as a versatile clinical tool capable of distilling diverse genetic variation into more readily interpretable transcriptional manifestations [5]. RNA-based profiling of genetic disease has traditionally occurred in targeted assays, with limited assessment of transcriptome-wide applications. Three recent studies reported on the utility of RNA-Seq as a complement to exome-based sequencing in inherited muscle pathologies [6], mitochondriopathies [7] and broad-spectrum rare disease [8]. Cummings *et al*. studied aberrant splicing patterns and allele-specific expression (ASE), achieving a diagnostic improvement of 35%, while Kremer *et al*. and Fresard *et al*. evaluated splicing, ASE, and gene expression quantification, increasing diagnostic yields by 10% and 7.5% respectively. These studies concluded that RNA-Seq represents an essential component of the diagnostic toolkit for rare genetic disease testing.

One transcriptional phenomenon not considered by these previous studies is the expression of fusion transcripts. This is the occurrence whereby genetic material from mutually distinct genes is aberrantly conjoined and transcribed. It can occur by translocation, inversion, deletion, and duplication, potentially leading to gained, lost or altered gene function. Human gene-fusion transcripts are known to occur in hematological and solid tissue cancers where their oncogenic, diagnostic and therapeutic relevance are well-documented [9]. However, the systematic application of fusion transcript detection in germline genetic disease is absent from the literature. This is despite the fact that mechanisms commonly responsible for fusion transcript formation, including deletions, inversions and translocations, often underlie inherited conditions [10]. Indeed, case studies have reported fusion transcripts in disease including brain malformation [11] [12] [13], intellectual disability [14] [15] [16] [17] [18], schizophrenia [19] [20], spastic paraplegia [21], autism spectrum disorder [22], Gille de la Tourette Syndrome [23] and more [24] [25] [10]. These sporadic cases suggest that the systematic inclusion of fusion transcript detection in RNA-based analysis of rare undiagnosed disease may lead to improved diagnostic rates.

Despite the availability of fusion-detection software, its practical application to transcriptome-wide rare disease studies in germline samples is challenging. Current solutions show limited agreement in the putative fusion candidates they output and none generate fully inclusive results. An appropriate fusion caller should be selected to match the data type under analysis. However, current tools were trained using cell line, tumor, or *in-silico* datasets and are not applicable to germline data. Filters empirically derived from mismatched training data lead to low sensitivity when profiling unrelated sample types [26]. Another obstacle to fusion detection in germline samples is the abundance of false-positive findings arising from bioinformatics alignment artifacts, PCR artifacts, DNA fragments or unprocessed mRNA [27]. Equally, the potential remains for the detection of genuine mRNA species, commonly originating from currently unrecognized single genes, or more rarely, from trans-splicing mechanisms [27–31]. Furthermore, non-pathogenic constitutive fusions may be detected [32] [30], or fusions occurring transiently in subclonal cell populations [33]. Thus, any attempt to systematically apply fusion transcript detection in inherited disease studies using germline samples will require methods to detect meaningful fusion candidates and deprioritize phenotypically inconsequential results.

Here, we describe the systematic application of fusion transcript detection to a cohort of 47 individuals with undiagnosed rare genetic disease. By applying a custom annotation and categorization process to fusion candidates, we demonstrate the presence of diagnostic fusion transcripts in a subset of patients. Our findings provide an analytical framework for others in the field and provide justification for the routine application of fusion transcript identification in genetic disease patients who eluded a diagnosis with existing assays.

## Materials and methods

### Ethical compliance

This study was approved by the Mayo Clinic institutional review board and all participants provided written informed consent for genetic testing.

### Study subjects

All patients were clinically referred to Mayo Clinic’s Center for Individualized Medicine, seeking genetic diagnosis of a suspected rare inherited disease. Patients and parents underwent genetic counselling and a full case history and family pedigree were constructed. Patients not fully diagnosed by exome sequencing were selected for whole-transcriptome RNA sequencing.

### RNA-sequencing

Sequencing was conducted on blood for 46 patients and cultured fibroblasts for 1 patient due to sample availability. Blood-derived RNA was obtained by collecting peripheral whole blood in PAXgene blood RNA tubes and using the QIAcube system (Qiagen) according to the manufacturer’s protocol for RNA extraction. RNA was isolated from fibroblasts as previously described [34].

Sequencing libraries were prepared with either the TruSeq RNA Sample Prep Kit v2 or the TruSeq RNA Access Library Prep Kit (Illumina, San Diego, CA). Paired-end 101-basepair reads were sequenced on an Illumina HiSeq 2500 using the TruSeq Rapid SBS sequencing kit version 1 and HCS version 2.0.12.0 data collection software. A median of approximately 200 million reads was generated per individual. Base calling was performed using Illumina’s RTA version 1.17.21.3.

### RNA fusion analysis

**Fig 1:**
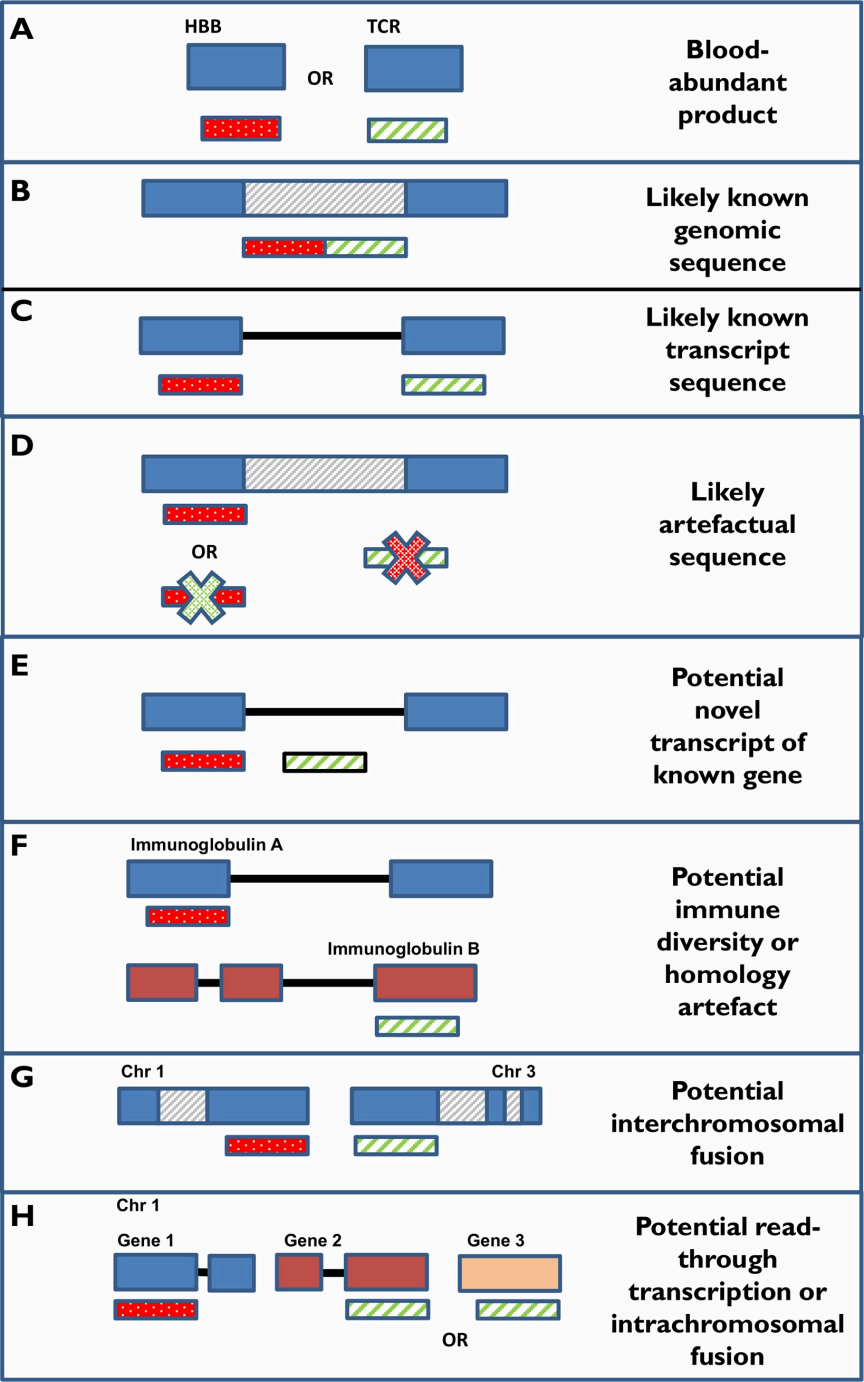
Fusion candidate BLAST categorization rationale. Putative fusion sequences were BLASTN aligned to the human genome and transcriptome to enable categorization. A) Candidates aligning to abundant hematological genes (Globins, T-cell receptors) were not considered further due to their overrepresentation in blood samples and observed overrepresentation in fusion analysis results. These might represent artifacts or transient biological events. B & C) Full length candidates producing unbroken alignments against the human transcriptome or genome were classified as likely known transcripts or genomic sequence respectively. D) When the candidate produced no alignment against the human genome or transcriptome or only a part alignment was possible, the candidate was classified as a likely artifact, potentially containing low quality or non-human sequence including adapters. E) When the candidate produced multiple alignments within the gene boundaries of a single gene but did not align completely to a known transcript, it was classified as a potential novel transcript of a known gene. This category has the also potential to capture aberrant single-gene events. F) When the candidate produced two hits to separate immunoglobulins the event was classed as potentially representing immune diversity. Alternatively these may be generated by alignment artifacts due to high homology between immunoglobulin genes. G) When two distinct alignments were produced against two different chromosomes, the candidate was defined as a potential interchromosomal fusion. Fused genes with known homology were flagged to enable additional checking for alignment artifacts. H) When the candidate aligned to two distinct genes or regions on a single chromosome, it was classified as a potential intrachromosomal fusion. Fused genes with known homology were flagged to enable additional checking for alignment artifacts. Intrachromosomal candidates occurring between neighboring genes were annotated as potential read-through events. These events could represent true fusions or aberrant transcriptional events but might also represent biologically normal events that occur due to co-transcription of neighboring genes that have yet to be re-classified as single genes. Interchromosomal and intrachromosomal candidates were annotated as *homologous* when the two hits occurred against known homologous genes based on the Duplicated Genes Database (http://dgd.genouest.org/). Such instances might represent artifacts due to misalignment between closely homologous genes or might equally represent true aberrant events, preferentially occurring due to homology at the genomic sequence level.

Candidate fusion events were initially detected using TopHat Fusion (TopHat release 2.1.0) [35]. Minimal depth filtering was applied to candidate fusions. Each fusion candidate was required to be supported by a single split read pair (one read-pair member mapping across the breakpoint) and a single spanning read pair (one read-pair member mapped to each side of the breakpoint). Ultimately this enabled us to maintain a strategy that was more inclusive than the default filters (3 split, 2 supporting) while still requiring supporting evidence from both classes of fusion-defining read pairs. To further increase candidate inclusiveness in this germline dataset, we omitted the cancer-cell-line-derived TopHat Fusion post-processing filter steps (tophat-fusion-post) and began with the unprocessed fusion calls as input into a candidate categorization workflow. We performed sequence alignment to the human genome and transcriptome using BLASTN (v2.6.0) [36]. A word size of 7 and e-value threshold of 1 was used to enable the BLAST alignment of short sequences. Alignments with less than 90% sequence identity and 75% sequence length coverage were filtered. Top scoring alignments were individually selected for (i) full length fusion candidates including conjoined 5`and 3`segments and (ii) decoupled 5`and 3`fusion candidate segments. Alignment results were annotated with Ensembl gene models [37] to identify putative gene involvement, exon-intron composition and coding-frame status, where applicable. Subsequent candidate classification rationale is detailed in Fig 1. Standard TopHat Fusion-filtered outputs were generated alongside custom categorized outputs to enable the comparison of results.

### Population frequency-based filtering

As our patient cohort suffered from rare disease, we assumed that any causative event would occur with extremely low frequency in a normal population. To control for event frequency and recurrent artifacts, we compared our putative fusion candidates to a fusion-event database generated using normal samples from our institution, the Illumina Human BodyMap and the Genotype-Tissue Expression (GTEx) project (dbGaP accession phs000424.v7.p2) [38] (approximately 11688 RNA-Seq samples from 500+ individuals and 53 tissue types in total). Most fusion candidates in normal controls were detected with only one supporting read (S1 Fig) and we theorized that the artefactual candidates were likely overrepresented close to this level of support. We therefore considered fewer than two supporting reads as insufficient evidence of a genuine fusion event in our control database. Putative fusion candidates were removed from consideration if they were identified more than two supporting reads in a normal control specimen. Candidates were not considered further if they appeared in another sample from our rare disease cohort, since the patients were unrelated and expected to suffer from rare and distinct genetic disorders.

### Phenotype-based prioritization of events classified as potential fusions

Putative fusion transcripts were evaluated with manual and automated approaches to ascertain potential relevance to each patient’s phenotype. The manual review of fusion transcripts was carried out to identify links to patient phenotype based on case notes, medical records, Online Mendelian Inheritance in Man (OMIM) [39], Genecards [40] and relevant literature. We also applied an automated *in-silico* method called PCAN: Phenotype consensus analysis to support disease-gene association [41] to predict the relevance of fusion-forming genes to phenotypes. PCAN uses semantic similarity scoring to measure relationships between the phenotypic terms mutually associated with a patient and a gene. Scores are ranked by simultaneously measuring semantic similarity for all disease-associated genes in the ClinVar database [42] versus each patient’s phenotype and producing a rank-score (rank/number of genes in Clinvar e.g. 0.01 indicates that a gene produces a score in the top 1% of all disease-linked ClinVar genes). PCAN also measures the phenotypic relevance of all genes sharing Reactome pathways [43] or STRING [44] protein-protein interaction networks with the fusion-forming genes, producing a p-value score and enabling indirect phenotypic-link discovery.

### Confirmation of fusion candidates

A selection of fusions passing filtering and phenotypic prioritization steps were selected for PCR validation. Fusion transcripts were amplified from cDNA generated from patient RNA using the Invitrogen Super-Script II RT Kit (Cat. No. 18064022) with random hexamer primers. PCR was performed with primers detailed in S1 File using Bioline MiTaq Polymerase (Cat. No. BIO-25043). Reaction conditions included an annealing temperature of 55°C for 30–34 cycles.

Droplet Droplet digital PCR (ddPCR) was also performed for all fusion sequences selected for validation. gBlock constructs (Integrated DNA Technologies) were synthesized as positive controls. ddPCR primers and gBlock sequences are described in S2 File. ddPCR reactions contained 11 μL of ddPCR EvaGreen Supermix (Bio-Rad), 2.2 μL of primer mix (100nM final concentration of each primer) and 8.8 μL of cDNA. Separate reactions were assembled for each fusion candidate using a corresponding primer set. The QX-100 Droplet Generator (Bio-Rad) generated droplets with 20 μL of sample mix and 70 μL of QX200 droplet generation oil Droplets were transferred to a semi-skirted plate and sealed at 180°C for 4 sec. Thermocycling conditions were as follows: enzyme activation at 95°C for 5 min, 40 cycles of denaturation at 95°C for 30 sec, annealing and extension at 60°C for 1 min, and signal stabilization at 4°C for 5 min and 90°C for 5 min. Plates were measured on a QX200 Droplet Reader (Bio-Rad).

Further validation work was performed for select fusion events. Agilent 44k and 180k array comparative genome hybridization (aCGH), fluorescence in-situ hybridization (FISH), multiplex-ligation probe analysis (MLPA) and Molecular Inversion Probe (MIP) Analysis were performed as previously described by Oliver *et al*. [45]. Flow cytometry, long range PCR, Pacific Biosciences (PacBio) sequencing, targeted PCR and Sanger sequencing were performed as previously described by Cousin *et al*. [34].

## Results

### Patient cohort

RNA-Seq was performed on 47 patients with an incomplete diagnosis following prior testing, including exome sequencing. The cohort consisted of 23 males and 24 females. Ages at initial referral ranged from 9 months to 68 years with a mean age of 18 years and median of 11 years. Clinical presentations varied widely and comprised a spectrum of neurological, immune, muscular, gastrointestinal, connective tissue and skeletal disorders (S1 Table).

### Genes of prior interest

Of 47 cases, 19 had genes or variants of potential interest identified by exome sequencing and clinical review (S2 Table). Two patients had variants or genes considered to be of exceptionally high interest. Patient 6 carried a single pathogenic variant in *ATM* with strong links to phenotype, but a second variant was required to fully explain the phenotype based on an autosomal recessive mode of inheritance. In patient 37, a pathogenic variant was actively sought in *EXT1* or *EXT2*. These genes of exceptionally high prior interest were determined to have expression levels suited to analysis in available tissue. Four further patients (Patients 21, 36, 42 and 44) carried variants with predicted pathogenicity and observed zygosity that was suspected to be fully explanatory of some element of their phenotype. It was theorized that fusion profiling for these patients might yield further phenotype-relevant events in other genes. The thirteen additional cases carried a selection of variants of unknown significance (VUS). Six of the thirteen patients carried a total of eight VUS in genes that displayed low expression (< 1 TPM in the GTEx [38] database) in whole blood, however, six of these showed correspondingly low expression in fibroblasts. Ultimately it was decided to proceed with sequencing of readily available blood samples for investigative purposes (S2 Table). The remaining 28 cases were unsolved and without candidates following exome sequencing, and were consequently included for exploratory analysis.

### Fusion candidate classification workflow

**Fig 2:**
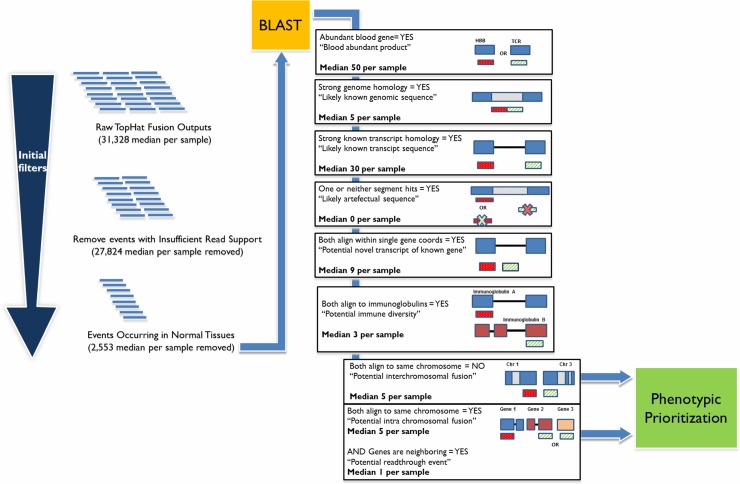
Fusion categorization workflow and median number of fusion candidates per category. Unfiltered results from TopHat fusion were BLASTed, annotated and input into the candidate classification workflow. The median number of events per sample in each category is shown. All candidates classified as potential fusions or read-through events, proceeded into a final review stage that determined phenotypic relevance of the genes to the patient condition using both automated PCAN analysis and manual review. Candidates classified as most phenotypically relevant were selected for follow-up validation.

The fusion candidate selection workflow with the median number of candidates per category is shown in Fig 2. This workflow was designed to remove suspected artifacts or recurrent fusions and to classify remaining candidates into biologically meaningful categories. The median number of unfiltered fusion candidates entering the workflow was 31,138 per patient. The minimal read-depth filter removed a median of 27,824 likely spurious events per patient. Removal of putative fusions previously observed in normal samples further reduced candidates by a median of 2,553 per patient. Remaining filtering and categorization steps reduced fusion candidates by a median value of 97, achieving a tractable median of 12 events per patient which were classified as potential fusions and subjected to manual review for links to phenotype. The number of candidates categorized per patient at each stage is detailed in S3 Table while all candidates classified as potential fusions are included in S4 Table. A total of 16 fusion candidates in 13 patients (including 1 reciprocal event) passed phenotypic review, with potential links between genes and phenotype identified based on a combination of PCAN analysis and manual curation (Table 1). Extended descriptions and rationale for inclusion of fusion candidates passing manual review are provided in S5 Table.

**Table 1:** Technical details of 16 fusion candidiates passing phenotypic review.

Patient ID	Fusion	Supporting vs Non-Supporting Reads	Fused at Exon boundaries?	Fusion preserves reading frame?	Inter/Intrachromosomal	Genomic coordinates (hg19)	Separation on chromosome (bp)	Transcripts	Strand	Detected by Standard TopHat Fusion Filters?
Patient 3	*ABCC2-CUTC*	10 vs 18	Exon-Exon	Yes	Intrachromosomal	chr10:101554225-chr10:101515382	38843	NM_000392 Exon 6—NM_015960 Exon 9	Forward–Forward	No
Patient 5	*NARS2-TENM4*	23 vs 22	Exon-Exon	No	Intrachromosomal	chr11:78239888-chr11:78369861	129973	NM_001243251 Exon 6—NM_001098816	Reverse-Reverse	No
Patient 6	*ATM-SLC35F2*	14 vs 6	Exon-Exon	Yes	Intrachromosomal	chr11:108129802-chr11:107663526	466276	NM_000051 Exon 16—NM_017515 Exon 8	Forward–Reverse	Yes
	*SLC35F2-ATM*	43 vs 2				chr11:107673727-chr11:108137898	464171	NM_017515 Exon 7—NM_000051 Exon 17	Reverse—Forward	
Patient 7	*NKAPD1-DLAT*	26 vs 33	Exon-Exon	No	Intrachromosomal	chr11:111951282-chr11:111907997	43285	NM_001301019 Exon 4—NM_001931 Exon 6	Forward—Forward	No
Patient 12	*C18orf32-DYM*	19 vs 5	Exon-Exon	No	Intrachromosomal	chr18:47009954-chr18:46956817	53137	NM_001199356 Exon 6—NM_017653 Exon 2	Reverse-Reverse	No
Patient 13	*SLC30A6-SPAST*	11 vs 22	Exon-Exon	Yes	Intrachromosomal	chr2:32409407-chr2:32340771	68636	NM_001330476 Exon 2—NM_199436 Exon 5	Forward-Forward	No
Patient 13	*UBR1-EPB42*	4 vs 2	Exon-Exon	No	Intrachromosomal	chr15:43398140-chr15:43489662	91522	NM_174916 Exon 1—NM_0001199 Exon 13	Reverse-Reverse	No
Patient 18	*ARL5A-NEB*	7 vs 3	Exon-Exon	No	Intrachromosomal	chr2:152659521-chr2:152590309	69212	NM_012097 Exon 6—NM_001271208 Exon 2	Reverse-Reverse	No
Patient 20	*TET3-DGUOK*	29 vs 44	Exon-Exon	Yes	Intrachromosomal	chr2:74230293 -chr2:74173846	56447	NM_001287491 Exon 2—NM_080916 Exon 3	Forward—Forward	No
Patient 21	*METTL22-ABAT*	15 vs 5	Exon-Exon	No	Intrachromosomal	chr16:8738582 -chr16:8829556	90974	NM_024109 Exon 10—NM_020686 Exon 2	Forward-Forward	No
Patient 33	*CACNB4-STAM2*	33 vs 12	Exon-Exon	No	Intrachromosomal	chr2:152954844 -chr2:153006743	51899	NM_000726 Exon 2—NM_005843 Exon 2	Reverse-Reverse	No
Patient 36	*CTSS-ARNT*	27 vs 21	Exon-Exon	Yes	Intrachromosomal	chr1:150737114 -chr1:150786715	49601	NM_001199739 Exon 2—NM_001668 Exon 20	Reverse—Reverse	No
Patient 36	*SON-FCRL3*	7 vs 45	Intron-Exon	No	Interchromosomal	chr21:34927578 -chr1:157670375	NA	NM_138927 Exon 3—NM_001320333 Exon 2	Reverse—Reverse	No
Patient 37	*PDPK1-PRSS21*	51 vs 120	Exon-Exon	No	Intrachromosomal	chr16:2633586 -chr16:2875971	242385	NM_002613 Exon 10—ENST00000575739.1 Exon 2	Forward—Forward	No
Patient 37	*SAMD12-EXT1*	17 vs 2	Exon-Exon	No	Intrachromosomal	chr8:119592952-chr8:118849438	743514	NM_001101676 Exon 2—NM_000127 Exon 2	Reverse-Reverse	No

Table 1 describes technical details of the fusion canddiates passing all steps of the categorization pipeline and putatively determined to have phenotypic relevance. Only one fusion candidate was detected by the standard Tophat Fusion filter settings.

### Confirmation of fusion candidates

Eleven candidate fusions with strong phenotypic relevance to the patient were selected for confirmation using orthogonal methods. Table 2 describes each of the fusions as well as the rationale for their selection and the status of their experimental confirmation. Eight fusions were successfully confirmed, with 2 clinically classified as diagnostic of the patients’ phenotype. Fusion confirmation images are included in S3 File. A selection of the confirmed fusion products are discussed in detail, as follows.

**Table 2:** Validation status and phenotypic justiifcation for the 11 fusion candidates selected for validation.

Patient ID	Fusion	Reason for interest?	Flagged by	Experimental Validation
Patient 5	*NARS2-TENM4*	Patient was referred due to epilepsy phenotype. NARS2 mutations are responsible for combined oxidative phospohorylation deficiency with symptoms including epilepsy. OMIM notes variable penetrance and severity.	PCAN (NARS2 reactome pathway p-value 0.027)	Positive (PCR, ddPCR)
Patient 6	*ATM-SLC35F2 (and SLC35F2-ATM)*	The patient carries a single pathogenic mutation in ATM, for which a second hit is sought as mutations are recessive.	Manual analysis & PCAN (ATM gene relative rank 0.002, Reactome pathway p-value 0.037, STRING p-value 0.028)	Positive (PCR, ddPCR, Sanger Sequencing, PacBio Sequencing)
Patient 12	*C18orf32-DYM*	Patient symptoms include microcephaly, global developmental delay and scoliosis. Mutations in DYM gene responsible for Dyggve-Melchior-Clausen disease whose symptoms include microcephaly, scoliosis, and psychomotor retardation.	PCAN (DYM gene relative rank 0.067, Reactome pathway, STRING p-value 0.028)	Positive (ddPCR)
Patient 13	*SLC30A6-SPAST*	Fusions between these two genes have been previously described in cases of spastic paraplegia. SPAST mutations are responsible for autosomal dominant spastic paraplegia (which the patient is not diagnosed with) but also various symptoms based on mutation e.g. mild-moderate cognitive defects, stutter, wheelchair bound by age 40 etc (OMIM).	Manual analysis.	Negative (PCR & ddPCR)
Patient 18	*ARL5A-NEB*	NEB mutations are responsible for Nemaline Myopathy. Symptoms include hypotonia and delayed motor development. Patient symptoms are developmental delay, hypotonia & laryngomalacia.	PCAN (NEB gene relative rank 0.03)	Positive (ddPCR)
Patient 20	*TET3-DGUOK*	TET3 is a TET Oncogene family member. TET3-DGUOK fusions have been reported in tumors.	Manual analysis	Negative (PCR & ddPCR)
Patient 33	*CACNB4-STAM2*	CACNB4 mutations are associated with episodic ataxia (inc. vertigo, nystagmus, dysarthria) and epilepsy. Patient phenotype is progressive gait difficulty/balance, abnormal brain MRI with atrophy, progressive cognitive decline.	Manual analysis. Overlap quite weak.	Negative (PCR & ddPCR)
Patient 36	*SON-FCRL3*	ZTTK syndrome is caused by haploinsufficiency of SON (AD inheritance). Symptoms include congenital heart defects, developmental delay, strabismus, various facial dysmorphisms, cleft palate. Patient has all of these plus a couple more.	Manual analysis	Positive (ddPCR)
Patient 37	*PDPK1-PRSS21*	Both genes fell at the boundaries of a deletion detected in this patient by aCGH. Links to phenotype remain unclear.	Manual analysis—phenotypic relevance unknown but corresponds to a deletion detected by aCGH.	Positive (PCR, ddPCR, aCGH, FISH)
Patient 37	*SAMD12-EXT1*	EXT1 mutations are known to cause many cases of multiple exostoses. Patient has unresolved exostoses.	Manual analysis & PCAN (EXT1 gene relative rank 0.001, Reactome p-value 0.00025, STRING p-value 0.0000066)	Positive (PCR, ddPCR, MIP, aCGH) Negative (MLPA, initial clinical aCGH)

Table 2 describes the 11 fusions selected for validation and phenotypic evidence putatively linking them to the patient phenotype. 8 of 11 fusions were successfully validated by orthogonal technologies. Validation status and utilized technologies are described.

### SAMD12-EXT1 fusion in a patient with multiple exostoses

**Fig 3:**
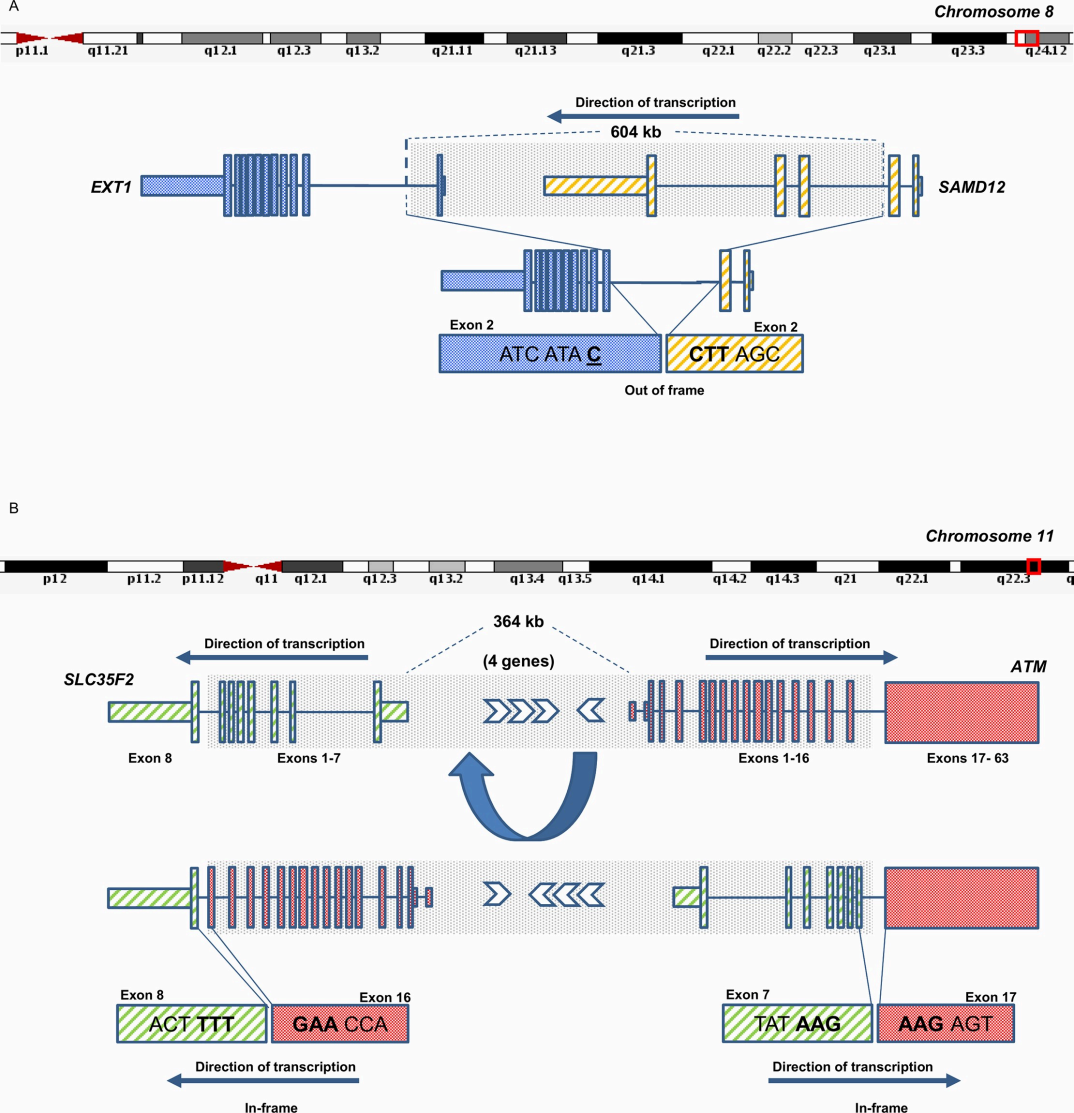
Diagnostic fusion transcripts identified by RNA-Seq in Mendelian disease cases. 3A) A *SAMD12-EXT1* fusion identified in Patient 37 whose phenotype included multiple exostoses. Multiple exostoses are most often attributed to autosomal dominant mutations in *EXT1* and *EXT2* but extensive clinical testing failed to identify any variants of interest in either gene. RNA-Seq identified a fusion candidate which might be explained by an interstitial deletion based on the genes’ orientation and position on chromosome 8 and would lead to loss of function of both *EXT1* and *SAMD12* due to loss of coding potential at the fusion boundary. Despite clinical aCGH and MLPA results initially indicating no deletion affecting the putatively conjoined genes, reevaluation of clinical aCGH results appeared suggestive of a mosaic deletion of approximately 604 kb at chr8:118960168–119569348. The deletion was subsequently validated by several orthogonal methods and determined to be diagnostic of the multiple exostoses phenotype. The *SAMD12-EXT1* fusion was not detected by standard TopHat filters. 3B) Reciprocal *ATM-SLC35F2* and *SLC35F2-ATM* fusions detected in Patient 6, with a severe combined immunodeficiency phenotype. The patient carried a paternally inherited pathogenic *ATM* variant for which a second hit was sought due to the autosomal recessive nature of *ATM* mutations. RNA-Seq revealed reciprocal fusions that were expected to retain their protein-coding potential but lead to aberrant *ATM* function based on the results of a novel flow cytometry assay. The fusions were experimentally validated by several orthogonal methods and shown to be maternally inherited, equating to compound heterozygous loss of *ATM* function which was classified as diagnostic of the patient phenotype. These reciprocal fusions were the only members of our validation panel that were detected by standard TopHat filters.

Patient 37 is a male child who presented with a phenotype including pachygyria, epilepsy, developmental delay, short stature, failure to thrive, facial dysmorphisms, and multiple exostoses [45]. Trio-based clinical exome sequencing identified a maternally inherited, X-linked loss-of-function variant in Doublecortin (*DCX*), which was classified as pathogenic and diagnostic of the patient’s neurological phenotype. However, the cause of the patient’s multiple exostoses remained unknown. Hereditary multiple exostoses is an autosomal dominant disorder, caused by pathogenic variants in *EXT1* or *EXT2* in 70–95% of cases, with *EXT1* affected twice as frequently as *EXT2* [46] [47]. Mosaic pathogenic events have been reported in numerous instances [48] [49]. No variant was identified in either gene despite extensive clinical testing, including array comparative genome hybridization (aCGH), metaphase karyotyping, multiplex ligation-dependent probe amplification (MLPA) and exome sequencing. RNA-Seq and subsequent fusion analysis discovered a candidate intrachromosomal fusion between *SAMD12* and *EXT1* (Fig 3A). The fusion was observed at the 3’ boundary of *SAMD12* exon 2 and the 5’ boundary of *EXT1* exon 2 forming a transcript predicted to be out-of-frame, leading to loss-of-function. The fusion was supported by 17 sequence reads and was not identified in our normal control database. *SAMD12* lies upstream of *EXT1* on Chromosome 8 and both genes are oriented on the reverse chromosomal strand. Intuitively, the fusion transcript could be expected to result from a rare interstitial deletion of genomic sequence between the two genes, however, prior clinical testing did not report this. The clinical aCGH results were re-inspected for evidence of a deletion in this region and a 604 kb genomic region intervening the fused exons (chr8:118960168–119569348) showed evidence of mosaic loss of *EXT1* exon 1 and *SAMD12* exons 3–5, but did not meet clinical-reporting thresholds. The mosaic loss was subsequently confirmed by an increased density aCGH (S2 Fig), MIP analysis (S3 Fig), PCR and ddPCR (S3 File). Thus, the *SAMD12-EXT1* fusion was categorized as pathogenic and diagnostic of the patient’s multiple exostoses phenotype in accordance with American College of Medical Genetics and Genomics (ACMG) reporting guidelines [50]. While exon 1 deletions are recurrently reported in cases of multiple exostoses, no previously reported events involve *SAMD12* or report fusion transcript formation [51].

### PDPK1-PRSS21: A patient carries a second confirmed fusion

A candidate *PDPK1-PRSS21* fusion was also identified in Patient 37, juxtaposing *PDPK1* Exon 10 and *PRSS21* Exon 2 at exon boundaries. The event was absent from our normal control database and aCGH revealed a corresponding 16p13.3 deletion spanning approximately 219 kb at chr16:2636111–2854742 (S4 Fig). The deleted interval completely contained ten genes, including *LOC652276*, *FLJ42627*, *ERVK13-1*, *KCTD5*, *PRSS27*, *SRRM2-AS1*, *SRRM2*, *TCEB2*, *PRSS33* and *PRSS41*, with *PDPK1* and *PRSS21* partially affected at the 5`and 3`boundaries respectively (S5 Fig). The *de novo* deletion was confirmed by FISH. None of the ten deleted genes had known links to patient phenotype. The *PRSS21* Ensembl transcript ENST00000575739.1 is a transcript of unknown function, not believed to include an open reading frame. *PDPK1* is a protein kinase implicated in cancer and a regulator of *CBP*. Pathogenic variants in *CBP* cause Rubinstein-Taybi Syndrome, an autosomal dominant condition. Whether the gene-fusion has phenotypic relevance to the patient is uncertain. The *DCX* variant and *SAMD12-EXT1* fusion are diagnostic of the majority of the patient’s phenotype, but further variation may still play a role in the broader phenotypic presentation. Ultimately the deletion and corresponding gene fusion constitute VUS that should be likely reevaluated over time as knowledge about the genes’ phenotypic relevance increases.

### Reciprocal ATM-SLC35F2 fusion in a patient with severe combined immunodeficiency

Patient 6 is a female infant diagnosed with T cell lymphopenia by newborn screening for severe combined immunodeficiency (SCID) [34]. SCID gene panel sequencing was uninformative and aCGH unrevealing. Subsequent trio-based exome sequencing discovered a paternally inherited frameshift INDEL in *ATM*, clinically classified as pathogenic. Pathogenic *ATM* variation causes Ataxia-telangiectasia in an autosomal recessive manner, and would account for the patient’s phenotype if a second variant was in *trans*. Flow cytometry assay revealed impaired phosphorylation of ATM, supporting the presence of a second pathogenic variant [34]. RNA sequencing of patient fibroblasts revealed reciprocal *ATM-SLC35F2* and *SLC35F2-ATM* fusion transcripts (Fig 3B). These fusions were supported by 14 and 43 reads respectively, and neither was identified in our normal control database. The *ATM-SLC35F2* fusion consists of *ATM* exon 16 joined to *SLC35F2* exon 8, while the *SLC35F2-ATM* fusion consists of *SLC35F2* exon 7 joined to *ATM* exon 17. Both resulting fusions were predicted to be in-frame, with each gene fragment in its correct orientation, despite the two genes existing natively on opposing genomic strands on Chromosome 11q22.3. It was hypothesized that the reciprocal fusion transcripts were the result of a chromosomal inversion. To confirm the hypothesis, long range PCR of the putatively affected introns was conducted and sequenced using PacBio long-read technology (S6 Fig). This resulted in reads bridging the breakpoints, which were subsequently confirmed by targeted PCR (S3 File) and Sanger sequencing (S7 Fig). The event was shown to be inherited from the unaffected mother, equating to a compound heterozygous loss of ATM function in the patient. Thus the event was classified as diagnostic of the patient’s phenotype in accordance with ACMG guidelines.

### Fusion selection by default TopHat Fusion filtering

The default TopHat Fusion filters identified a total of 1003 candidates in our patient cohort (5–46 per patient). We classified these candidates using our categorization workflow (S6 Table). 52.3% of candidates involved blood-abundant genes while a further 19.7% involved immunoglobulin genes. The majority of candidates (994 of 1002) were removed due to their presence in our normal control database (S7 Table). All candidates detected by TopHat Fusion’s default filters and classified by our workflow as potential fusions are described in S8 Table irrespective of normal tissue expression. Candidates occurring in normal tissue databases but categorized as potential fusions included known polymorphic events such as *KANSL1-ARL17A/B* [52] and *TFG-GPR128* [53], detected in 14 and 3 patients respectively. Other events such as *PFKFB3-RP11#563J2*.*2* (37 patients) and *EIF4E3-FOXP1* (28 patients) appeared with high frequency and might represent previously unrecognized polymorphic fusion events or read-through transcription. The 9 remaining candidates not appearing in our normal database comprised 3 containing blood-abundant genes, 1 potential novel transcript and 5 events categorized as potential fusions (two representing the reciprocal *ATM* fusion). Thus 99.5% of the standard TopHat Fusion outputs were removed from further consideration by our classification workflow. Of the five mutually detected fusion candidates, the reciprocal *ATM* fusions were the only ones selected by our categorization and prioritization workflow (Table 1). The remaining three fusion candidates were excluded from our manual analysis due to lack of phenotypic relevance. Within the group of 16 phenotypically prioritized fusion candidates output by our workflow, 8 of 11 attempted were successfully validated and only 2 were detected by the default TopHat Fusion filters.

## Discussion

We have described the first systematic application of fusion transcript detection in an undiagnosed, rare inherited disease cohort. Our findings support the assertion that fusion transcription is a phenomenon whose pathogenic relevance extends beyond the traditionally recognized field of oncology, and furthermore, suggest that fusion analysis is an important component of comprehensive rare inherited disease testing. The two confirmed diagnostic fusions reported here involve genes that were previously suspected of clinical significance but for which a pathogenic event was still sought following clinical and research testing using several advanced methods. The fact that fusion analysis achieved diagnosis where multiple alternative methods failed underscores the diagnostic potential of fusion profiling in rare disease cases. We assert that fusion analysis should be considered integral to any RNA-Seq pipeline used for genetic diagnosis.

The discovery of *SAMD12-EXT1* and reciprocal *ATM-SLC35F2* fusions constitutes a 4.3% increase in diagnostic yield within our patient cohort. Notably, the diagnostic odyssey cases studied here represent a phenotypically diverse and challenging population, and it cannot be discounted that similar analyses might produce higher rates of diagnosis within distinct phenotypic groupings. The clinical significance of the 5 additionally validated fusions remains unknown despite experimental verification and the potential phenotypic relevance of their constituent genes. The *EXT1* and *ATM* fusions are unique in that they affect genes with extensive prior evidence linking them incontrovertibly to each patient’s phenotype. The events containing genes with lesser-evidenced links to patient phenotype are challenging to conclusively interpret and consequently these remain variants of uncertain significance. It is possible that periodic reassessment of such events will eventually identify a pathogenic role as knowledge in the field expands. Alternatively, functional validation studies remain an available but non-trivial option to clarify the role of such fusions.

We developed an inherited-disease-focused workflow to replace fusion-filtering strategies developed for alternative applications, and to lower the potential for erroneous removal of disease-relevant events while reducing an initially overwhelming number of fusion calls to a tractable quantity. Thus our workflow provides a call set that is amenable to manual analysis and interpretation in Mendelian disease studies. Furthermore all events detected by the default TopHat Fusion filters were detected by our workflow, but were biologically classified and largely deprioritized following sequence alignment and biological inference. Conversely, of the 16 fusion candidates prioritized by our workflow, 73% of those tested were experimentally validated and only one reciprocal fusion was detected by the standard TopHat Fusion filters.

Initial raw candidate identification remains wholly dependent on the underlying fusion calling algorithm, and suitable care in its selection is required. We selected TopHat Fusion based on its ability to provide output of unfiltered candidate fusions. While this approach proved effective in this study, an ensemble of multiple callers might enable the detection of additional fusion events and represents a natural extension of our approach that should be considered in future studies.

The rationale underlying our candidate categorization workflow is versatile and widely applicable. Its various components can be implemented wholly or piecemeal, as part of new or existing workflows utilizing a wide range of fusion calling algorithms. For example, we demonstrated the ability to remove events likely to have low phenotypic relevance from the outputs of standard fusion-caller filters as evidenced by our reduction of the default TopHat Fusion outputs from a median of 19 events to less than a single event per patient. Furthermore, we have demonstrated that comparison to normal tissue databases alone will markedly reduce the number of candidates of unlikely phenotypic relevance.

This study reveals that surrogate tissues, such as blood, are viable biospecimens for the profiling of fusion transcription in inherited disease studies. Inaccessibility of affected tissue is a recognized obstacle to RNA-Seq profiling because of tissue-specific gene expression and splicing patterns [6] [7], therefore the successful utilization of surrogate tissue sources for fusion detection is encouraging. Nonetheless, this approach poses challenges and constraints that should not be overlooked. While approximately 68% of OMIM genes are expressed in fibroblasts for example [7], the genes underlying muscle pathologies are underrepresented in both fibroblasts and blood [6]. Within our own cohort, several genes of potential interest were scarcely expressed in either blood or fibroblasts, and we cannot discount the possibility that our analyses may have failed to detect pathogenic events in these under-expressed genes. Thus, inaccessibility of affected tissue may limit the utility of RNA-based approaches and the viability of these methodologies may require assessment on a case-by-case basis.

Conversely, the direct profiling of disease-affected tissue may represent its own challenges. Our findings indicate that genes highly expressed in blood are a major source of transcriptional or artifactual noise, and whilst it is convenient to remove blood-abundant genes from an analysis unrelated to blood pathologies, it will be less viable to remove genes highly expressed in muscle if directly profiling the affected tissue for the underlying cause of a muscular phenotype. Illustratively, Patient 6 was the only case for which fibroblasts were utilized in our study, and a correspondingly large number of candidate fusion events were categorized, often involving highly expressed species such as collagens. It is likely that some customization of normal tissue databases and excluded gene lists will be required to enable adequate categorization of common tissue-specific normal events or artifacts. Ideally, large scale multi-tissue sequence analysis efforts like GTEx will multiply and broaden to increase the sampled population and include protocols like fusion transcript analysis, thus facilitating continued and expanded analyses like our own.

Automated PCAN analysis ranked our two diagnostic fusions highest (rank scores 0.001 & 0.002) and flagged 8 of our final 16 candidates in total, raising the possibility of a workflow without a requirement for manual candidate prioritization. Nonetheless, technical errors remain a reality, and PCR or other confirmation studies are necessary to confirm a candidate’s presence. While our unsuccessfully validated fusions might represent an assortment of artifactual species, it is notable that all but one of them fused precisely at exon-exon boundaries, consistent with RNA splicing, and further they produced non-promiscuous alignments to the human genome and transcriptome. Furthermore, fusions between *SPAST* and *SLC30A6* as reported in Patient 13 have been previously reported in disease [54]. Such observations raise some uncertainty about the artifactual origins of these candidates. Alternative possibilities include the presence of low copy-number events due to mosaicism, subclonality, tissue-specific gene expression, or other novel RNA rearrangements, and thus, validation efforts utilizing alternative tissue sources might represent a means of categorizing putative artifactual events with more certainty.

Since both diagnostic events identified in this study result from underlying genomic deletion or rearrangement, the question arises of whether whole-genome sequencing could detect them. Without further analysis, the possibility cannot be discounted. Whole-genome analysis nonetheless brings its own set of analytical and interpretive problems. DNA does not match the ability of RNA to measure transcriptional consequence [6], [5], [4] and has its own technical limitations that may cause failure to detect chromosomal DNA fusions [27]. We believe that whole genome analyses will indubitably play a major role in the increased diagnosis of rare disorders as it spreads in use and its complexities are further unraveled, but ultimately DNA and RNA-based methods will serve as supplementary and parallel methodologies.

We focus primarily on DNA-Seq and RNA-Seq because they represent the most mature modern ‘omics’ technologies and the two that are being most widely applied in the rare disease domain. However, alternative approaches including those that integrate proteomic-based technologies also have the potential to detect aberrant fusion events. Throughput is currently higher with RNA-based methods, enabling more rapid, extensive and cost-effective profiling. Furthermore, fusion transcripts may or may not produce a protein product depending on their constitution. For example, an out-of-frame fusion leading to loss-of-function of two genes would not be expected to produce a protein. Thus RNA-Seq offers advantages of detectability beyond that of protein based assays, however proteomic and other approaches including diverse multi-omic assays will likely reveal their own benefits in the future as they become more accessible and their use becomes more ubiquitous.

While this study has focused on the detection of aberrant fusion transcripts, further diagnoses may yet be possible by expanding testing to include profiling of ASE, aberrant expression levels and splicing [6–8]. Indeed, we have previously published case studies where such events were diagnostic of rare disease [55]. Furthermore, variations of the analytical approach described herein may yield further events of interest. For example, the event category housing potential novel transcripts from single genes might contain abnormal exon combinations arising from intragenic deletions and these have potential for disease relevance. Ultimately however, each of these analyses is methodologically distinct and forms its own set of technical challenges. Their systematic application to this and further patient cohorts should undoubtedly form the basis of future work.

## Conclusions

We have reported the first successful systematic application of fusion transcript detection within a rare disease cohort. We have demonstrated an increased diagnostic rate and identified further novel candidates for phenotype causation. Fusion transcript analysis such as those described herein should be considered in any RNA-Seq analysis aimed at genetic diagnosis of undiagnosed rare inherited disease.

## Supporting information

S1 Table Demographic and phenotypic details of patient cohort. (XLSX)

S2 Table Prior identified events of interest in the patient cohort and gene expression levels in normal tissue. (XLSX)

S3 Table Number of events in each categorization grouping for all patients. (XLSX)

S4 Table All events categorized as fusion candidates prior to phenoytypic prioritization. (XLSX)

S5 Table Extended description of all fusion events passing the phenotypic prioritization step. (XLSX)

S6 Table Categorization of candidates passing standard the Tophat Fusion filters. (XLSX)

S7 Table Custom pipeline categorization of all events passing standard TopHat Fusion filters. (XLSX)

S8 Table Description/counts of fusions passing standard TopHat Fusion filters regardless of presence in normal database. (XLSX)

S1 Fig Histogram showing number of supporting reads per putative fusion event detected in the GTEx normal tissue RNA database. (PPTX)

S2 Fig Validation of the mosaic deletion underlying the *SAMD12-EXT1* fusion in patient 37. Despite initially negative clinical aCGH findings (Agilent 44k array), re-evaluation of sub calling threshold results suggested the presence of a mosaic deletion that was subsequently confirmed by increased density Agilent 180k array.
(PPTX)

S3 Fig Molecular inversion probe analysis showing deletion of *EXT1* exon 1 in patient 37. (PPTX)

S4 Fig 16p13.3 deletion detected by clinical aCGH in Patient 37. Reduced probe intensities and associated genes are demarcated by the red outline. PDPK1 and PRSS21 are seen at the boundaries.
(PPTX)

S5 Fig A 16p13.3 deletion creates a PDPK1-PRSS21 fusion in Patient 37. The deleted interval contained 10 genes with PDPK1 and PRSS21 lying at the 5’ and 3’ boundaries respectively. While a link to patient phenotype cannot be ruled out, the relevance of the deletion and fusion remain uncertain in the light of the co-occurring *SAMD12-EXT1* fusion and *DCX* variant which were both classified as pathogenic.
(PPTX)

S6 Fig Screenshot of raw sequencing reads from Patient 6’s PacBio sequencing of long-range PCR spanning from *SLC35F2* exon 7 to *ATM* exon 17 (3.5 kb product). Reads are shown aligned to the fused sequence in window showing the breakpoint in *SLC35F2* intron 7 and *ATM* intron 16.
(PPTX)

S7 Fig Chromatogram of Sanger sequenced Patient 6 PCR product showing mother and proband share the chromosome 11 inversion causative of the reciprocal *ATM-SLC35F2* fusion. (PPTX)

S1 File Primers used in PCR validation of fusion candidates. (DOCX)

S2 File Primers used in ddPCR validation of fusion candidates. (DOCX)

S3 File Raw TopHat Fusion outputs for Patients 1–5 and 7–10. (TAR)

S4 File Raw TopHat Fusion outputs for Patient 6. (TAR)

S5 File Raw TopHat Fusion outputs for Patients 11–19. (TAR)

S6 File Raw TopHat Fusion outputs for Patients 20–29. (TAR)

S7 File Raw TopHat Fusion outputs for Patients 30–39. (TAR)

S8 File Raw TopHat Fusion outputs for Patients 40–47. (TAR)

## References

[1] Sawyer SL, Hartley T, Dyment DA, Beaulieu CL, Schwartzentruber J, Smith A, et al Utility of whole-exome sequencing for those near the end of the diagnostic odyssey: time to address gaps in care. Clin Genet. 2016;89(3):275–84. 10.1111/cge.12654 26283276 PMC5053223

[2] Posey JE, Rosenfeld JA, James RA, Bainbridge M, Niu Z, Wang X, et al Molecular diagnostic experience of whole-exome sequencing in adult patients. Genetics in medicine: official journal of the American College of Medical Genetics. 2016;18(7):678–85. 10.1038/gim.2015.142 PMC4892996 26633545

[3] Yang YP, Muzny DM, Reid JG, Bainbridge MN, Willis A, Ward PA, et al Clinical Whole-Exome Sequencing for the Diagnosis of Mendelian Disorders. New Engl J Med. 2013;369(16):1502–11. 10.1056/NEJMoa1306555 24088041 PMC4211433

[4] Kremer LS, Wortmann SB, Prokisch H. "Transcriptomics": molecular diagnosis of inborn errors of metabolism via RNA-sequencing. J Inherit Metab Dis. 2018;41(3):525–32. 10.1007/s10545-017-0133-4 29372369 PMC5959960

[5] Byron SA, Van Keuren-Jensen KR, Engelthaler DM, Carpten JD, Craig DW. Translating RNA sequencing into clinical diagnostics: opportunities and challenges. Nat Rev Genet. 2016;17(5):257–71. 10.1038/nrg.2016.10 26996076 PMC7097555

[6] Cummings BB, Marshall JL, Tukiainen T, Lek M, Donkervoort S, Foley AR, et al Improving genetic diagnosis in Mendelian disease with transcriptome sequencing. Sci Transl Med. 2017;9(386). 10.1126/scitranslmed.aal5209 PMC5548421 28424332

[7] Kremer LS, Bader DM, Mertes C, Kopajtich R, Pichler G, Iuso A, et al Genetic diagnosis of Mendelian disorders via RNA sequencing. Nat Commun. 2017;8 10.1038/s41467-017-00021-9 28604674 PMC5499207

[8] Fresard L, Smail C, Ferraro NM, Teran NA, Li X, Smith KS, et al Identification of rare-disease genes using blood transcriptome sequencing and large control cohorts. Nat Med. 2019;25(6):911–9. 10.1038/s41591-019-0457-8 31160820 PMC6634302

[9] Dai X, Theobard R, Cheng H, Xing M, Zhang J. Fusion genes: A promising tool combating against cancer. Biochim Biophys Acta Rev Cancer. 2018;1869(2):149–60. 10.1016/j.bbcan.2017.12.003 29357299

[10] van Heesch S, Simonis M, van Roosmalen MJ, Pillalamarri V, Brand H, Kuijk EW, et al Genomic and Functional Overlap between Somatic and Germline Chromosomal Rearrangements. Cell Rep. 2014;9(6):2001–10. 10.1016/j.celrep.2014.11.022 25497101

[11] Nothwang HG, Kim HG, Aoki J, Geisterfer M, Kubart S, Wegner RD, et al Functional hemizygosity of PAFAH1B3 due to a PAFAH1B3-CLK2 fusion gene in a female with mental retardation, ataxia and atrophy of the brain. Hum Mol Genet. 2001;10(8):797–806. 10.1093/hmg/10.8.797 11285245

[12] Ramocki MB, Dowling J, Grinberg I, Kimonis VE, Cardoso C, Gross A, et al Reciprocal fusion transcripts of two novel Zn-finger genes in a female with absence of the corpus callosum, ocular colobomas and a balanced translocation between chromosomes 2p24 and 9q32. European Journal of Human Genetics. 2003;11(7):527–34. 10.1038/sj.ejhg.5200995 12825074

[13] Di Gregorio E, Bianchi FT, Schiavi A, Chiotto AM, Rolando M, Verdun di Cantogno L, et al A de novo X;8 translocation creates a PTK2-THOC2 gene fusion with THOC2 expression knockdown in a patient with psychomotor retardation and congenital cerebellar hypoplasia. J Med Genet. 2013;50(8):543–51. 10.1136/jmedgenet-2013-101542 23749989 PMC4133931

[14] Yue Y, Grossmann B, Holder SE, Haaf T. De novo t(7;10)(q33;q23) translocation and closely juxtaposed microdeletion in a patient with macrocephaly and developmental delay. Hum Genet. 2005;117(1):1–8. 10.1007/s00439-005-1273-4 15834588

[15] Backx L, Seuntjens E, Devriendt K, Vermeesch J, Van Esch H. A balanced translocation t(6;14)(q25.3;q13.2) leading to reciprocal fusion transcripts in a patient with intellectual disability and agenesis of corpus callosum. Chromosome Res. 2011;19:S59–S. 10.1159/000321577 21042007

[16] Hackmann K, Matko S, Gerlach EM, von der Hagen M, Klink B, Schrock E, et al Partial deletion of GLRB and GRIA2 in a patient with intellectual disability. European Journal of Human Genetics. 2013;21(1):112–4. 10.1038/ejhg.2012.97 22669415 PMC3522202

[17] Moyses-Oliveira M, Guilherme RS, Meloni VA, Di Battista A, de Mello CB, Bragagnolo S, et al X-linked intellectual disability related genes disrupted by balanced X-autosome translocations. Am J Med Genet B. 2015;168(8):669–77. 10.1002/ajmg.b.32355 26290131

[18] Mayo S, Monfort S, Rosello M, Orellana C, Oltra S, Caro-Llopis A, et al Chimeric Genes in Deletions and Duplications Associated with Intellectual Disability. Int J Genomics. 2017;2017:4798474 10.1155/2017/4798474 28630856 PMC5463148

[19] Zhou X, Chen Q, Schaukowitch K, Kelsoe JR, Geyer MA. Insoluble DISC1-Boymaw fusion proteins generated by DISC1 translocation. Mol Psychiatry. 2010;15(7):669–72. 10.1038/mp.2009.127 20351725 PMC2891102

[20] Rippey C, Walsh T, Gulsuner S, Brodsky M, Nord AS, Gasperini M, et al Formation of chimeric genes by copy-number variation as a mutational mechanism in schizophrenia. Am J Hum Genet. 2013;93(4):697–710. 10.1016/j.ajhg.2013.09.004 24094746 PMC3791253

[21] Boone PM, Yuan B, Campbell IM, Scull JC, Withers MA, Baggett BC, et al The Alu-rich genomic architecture of SPAST predisposes to diverse and functionally distinct disease-associated CNV alleles. Am J Hum Genet. 2014;95(2):143–61. 10.1016/j.ajhg.2014.06.014 25065914 PMC4129405

[22] Ceroni F, Sagar A, Simpson NH, Gawthrope AJ, Newbury DF, Pinto D, et al A deletion involving CD38 and BST1 results in a fusion transcript in a patient with autism and asthma. Autism Res. 2014;7(2):254–63. 10.1002/aur.1365 24634087 PMC4309371

[23] Bertelsen B, Melchior L, Jensen LR, Groth C, Nazaryan L, Debes NM, et al A t(3;9)(q25.1;q34.3) translocation leading to OLFM1 fusion transcripts in Gilles de la Tourette syndrome, OCD and ADHD. Psychiatry Res. 2015;225(3):268–75. 10.1016/j.psychres.2014.12.028 25595337

[24] Mansouri MR, Carlsson B, Davey E, Nordenskjold A, Wester T, Anneren G, et al Molecular genetic analysis of a de novo balanced translocation t(6;17)(p21.31;q11.2) associated with hypospadias and anorectal malformation. Hum Genet. 2006;119(1–2):162–8. 10.1007/s00439-005-0122-9 16395596

[25] Borsani G, Piovani G, Zoppi N, Bertini V, Bini R, Notarangelo L, et al Cytogenetic and molecular characterization of a de-novo t(2p;7p) translocation involving TNS3 and EXOC6B genes in a boy with a complex syndromic phenotype. Eur J Med Genet. 2008;51(4):292–302. 10.1016/j.ejmg.2008.02.006 18424204

[26] Kumar S, Vo AD, Qin FJ, Li H. Comparative assessment of methods for the fusion transcripts detection from RNA-Seq data. Sci Rep-Uk. 2016;6. 10.1038/srep21597 PMC4748267 26862001

[27] Peng ZY, Yuan CF, Zellmer L, Liu SQ, Xu NZ, Liao DJ. Hypothesis: Artifacts, Including Spurious Chimeric RNAs with a Short Homologous Sequence, Caused by Consecutive Reverse Transcriptions and Endogenous Random Primers. J Cancer. 2015;6(6):555–67. 10.7150/jca.11997 26000048 PMC4439942

[28] Akiva P, Toporik A, Edelheit S, Peretz Y, Diber A, Shemesh R, et al Transcription-mediated gene fusion in the human genome. Genome Res. 2006;16(1):30–6. 10.1101/gr.4137606 16344562 PMC1356126

[29] He Y, Yuan C, Chen L, Lei M, Zellmer L, Huang H, et al Transcriptional-Readthrough RNAs Reflect the Phenomenon of "A Gene Contains Gene(s)" or "Gene(s) within a Gene" in the Human Genome, and Thus Are Not Chimeric RNAs. Genes (Basel). 2018;9(1). 10.3390/genes9010040 PMC5793191 29337901

[30] Yuan C, Han Y, Zellmer L, Yang W, Guan Z, Yu W, et al It Is Imperative to Establish a Pellucid Definition of Chimeric RNA and to Clear Up a Lot of Confusion in the Relevant Research. Int J Mol Sci. 2017;18(4). 10.3390/ijms18040714 PMC5412300 28350330

[31] Babiceanu M, Qin FJ, Xie ZQ, Jia YM, Lopez K, Janus N, et al Recurrent chimeric fusion RNAs in non-cancer tissues and cells. Nucleic Acids Res. 2016;44(6):2859–72. 10.1093/nar/gkw032 26837576 PMC4824105

[32] Aigner J, Villatoro S, Rabionet R, Roquer J, Jimenez-Conde J, Marti E, et al A common 56-kilobase deletion in a primate-specific segmental duplication creates a novel butyrophilin-like protein. Bmc Genet. 2013;14:61 10.1186/1471-2156-14-61 23829304 PMC3729544

[33] Yuan H, Qin F, Movassagh M, Park H, Golden W, Xie Z, et al A chimeric RNA characteristic of rhabdomyosarcoma in normal myogenesis process. Cancer Discov. 2013;3(12):1394–403. 10.1158/2159-8290.CD-13-0186 24089019

[34] Cousin MA, Smith MJ, Sigafoos AN, Jin JJ, Murphree MI, Boczek NJ, et al Utility of DNA, RNA, Protein, and Functional Approaches to Solve Cryptic Immunodeficiencies. J Clin Immunol. 2018;38(3):307–19. 10.1007/s10875-018-0499-6 29671115

[35] Kim D, Salzberg SL. TopHat-Fusion: an algorithm for discovery of novel fusion transcripts. Genome Biol. 2011;12(8). 10.1186/gb-2011-12-8-r72 PMC3245612 21835007

[36] Altschul SF, Gish W, Miller W, Myers EW, Lipman DJ. Basic Local Alignment Search Tool. J Mol Biol. 1990;215(3):403–10. 10.1016/S0022-2836(05)80360-2 2231712

[37] Zerbino DR, Achuthan P, Akanni W, Amode MR, Barrell D, Bhai J, et al Ensembl 2018. Nucleic Acids Res. 2018;46(D1):D754–D61. 10.1093/nar/gkx1098 29155950 PMC5753206

[38] Carithers LJ, Ardlie K, Barcus M, Branton PA, Britton A, Buia SA, et al A Novel Approach to High-Quality Postmortem Tissue Procurement: The GTEx Project. Biopreserv Biobank. 2015;13(5):311–9. 10.1089/bio.2015.0032 26484571 PMC4675181

[39] Hamosh A, Scott AF, Amberger J, Bocchini C, Valle D, McKusick VA. Online Mendelian Inheritance in Man (OMIM), a knowledgebase of human genes and genetic disorders. Nucleic Acids Res. 2002;30(1):52–5. 10.1093/nar/30.1.52 11752252 PMC99152

[40] Stelzer G, Rosen N, Plaschkes I, Zimmerman S, Twik M, Fishilevich S, et al The GeneCards Suite: From Gene Data Mining to Disease Genome Sequence Analyses. Curr Protoc Bioinformatics. 2016;54:1 30 1–1 3. 10.1002/cpbi.5 27322403

[41] Godard P, Page M. PCAN: phenotype consensus analysis to support disease-gene association. BMC Bioinformatics. 2016;17(1):518 10.1186/s12859-016-1401-2 27923364 PMC5142268

[42] Landrum MJ, Lee JM, Benson M, Brown G, Chao C, Chitipiralla S, et al ClinVar: public archive of interpretations of clinically relevant variants. Nucleic Acids Res. 2016;44(D1):D862–D8. 10.1093/nar/gkv1222 26582918 PMC4702865

[43] Fabregat A, Jupe S, Matthews L, Sidiropoulos K, Gillespie M, Garapati P, et al The Reactome Pathway Knowledgebase. Nucleic Acids Res. 2018;46(D1):D649–D55. 10.1093/nar/gkx1132 29145629 PMC5753187

[44] Szklarczyk D, Morris JH, Cook H, Kuhn M, Wyder S, Simonovic M, et al The STRING database in 2017: quality-controlled protein-protein association networks, made broadly accessible. Nucleic Acids Res. 2017;45(D1):D362–D8. 10.1093/nar/gkw937 27924014 PMC5210637

[45] Oliver GR, Blackburn PR, Ellingson MS, Conboy E, Pinto EVF, Webley M, et al RNA-Seq detects a SAMD12-EXT1 fusion transcript and leads to the discovery of an EXT1 deletion in a child with multiple osteochondromas. Mol Genet Genomic Med. 2019:e00560 10.1002/mgg3.560 30632316 PMC6418362

[46] Philippe C, Porter DE, Emerton ME, Wells DE, Simpson AH, Monaco AP. Mutation screening of the EXT1 and EXT2 genes in patients with hereditary multiple exostoses. Am J Hum Genet. 1997;61(3):520–8. 10.1086/515505 9326317 PMC1715939

[47] Wuyts W, Van Hul W. Molecular basis of multiple exostoses: mutations in the EXT1 and EXT2 genes. Hum Mutat. 2000;15(3):220–7. 10.1002/(SICI)1098-1004(200003)15:3<220::AID-HUMU2>3.0.CO;2-K 10679937

[48] Szuhai K, Jennes I, de Jong D, Bovee JV, Wiweger M, Wuyts W, et al Tiling resolution array-CGH shows that somatic mosaic deletion of the EXT gene is causative in EXT gene mutation negative multiple osteochondromas patients. Hum Mutat. 2011;32(2):E2036–49. 10.1002/humu.21423 21280143

[49] Sarrion P, Sangorrin A, Urreizti R, Delgado A, Artuch R, Martorell L, et al Mutations in the EXT1 and EXT2 genes in Spanish patients with multiple osteochondromas. Sci Rep-Uk. 2013;3. 10.1038/srep01346 PMC3581825 23439489

[50] Richards S, Aziz N, Bale S, Bick D, Das S, Gastier-Foster J, et al Standards and guidelines for the interpretation of sequence variants: a joint consensus recommendation of the American College of Medical Genetics and Genomics and the Association for Molecular Pathology. Genet Med. 2015;17(5):405–24. 10.1038/gim.2015.30 25741868 PMC4544753

[51] Jennes I, Pedrini E, Zuntini M, Mordenti M, Balkassmi S, Asteggiano CG, et al Multiple osteochondromas: mutation update and description of the multiple osteochondromas mutation database (MOdb). Hum Mutat. 2009;30(12):1620–7. 10.1002/humu.21123 19810120

[52] Boettger LM, Handsaker RE, Zody MC, McCarroll SA. Structural haplotypes and recent evolution of the human 17q21.31 region. Nat Genet. 2012;44(8):881–5. 10.1038/ng.2334 22751096 PMC4020351

[53] Chase J, Fiebig A, Ernst T, Grand F, Reiter A, Erben P, et al A Polymorphic Constitutional Tfg-Gpr128 Fusion in Healthy Individuals Identified by Targeted Array Cgh. Haematol-Hematol J. 2009;94:218–.

[54] Boone PM, Yuan B, Campbell IM, Scul JC, Withers MA, Baggett BC, et al The Alu-Rich Genomic Architecture of SPAST Predisposes to Diverse and Functionally Distinct Disease-Associated CNV Alleles. Am J Hum Genet. 2014;95(2):143–61. 10.1016/j.ajhg.2014.06.014 25065914 PMC4129405

[55] Boczek NJ, Hopp K, Benoit L, Kraft D, Cousin MA, Blackburn PR, et al Characterization of three ciliopathy pedigrees expands the phenotype associated with biallelic C2CD3 variants. Eur J Hum Genet. 2018;26(12):1797–809. 10.1038/s41431-018-0222-3 30097616 PMC6244354

