# Supplementary figures and images for: A tailored approach to fusion transcript identification increases diagnosis of rare inherited disease
Source: PLoS One. 2019 Oct 2;14(10):e0223337. doi: 10.1371/journal.pone.0223337 (PMC6774566; doi:10.1371/journal.pone.0223337)

## Slide 1
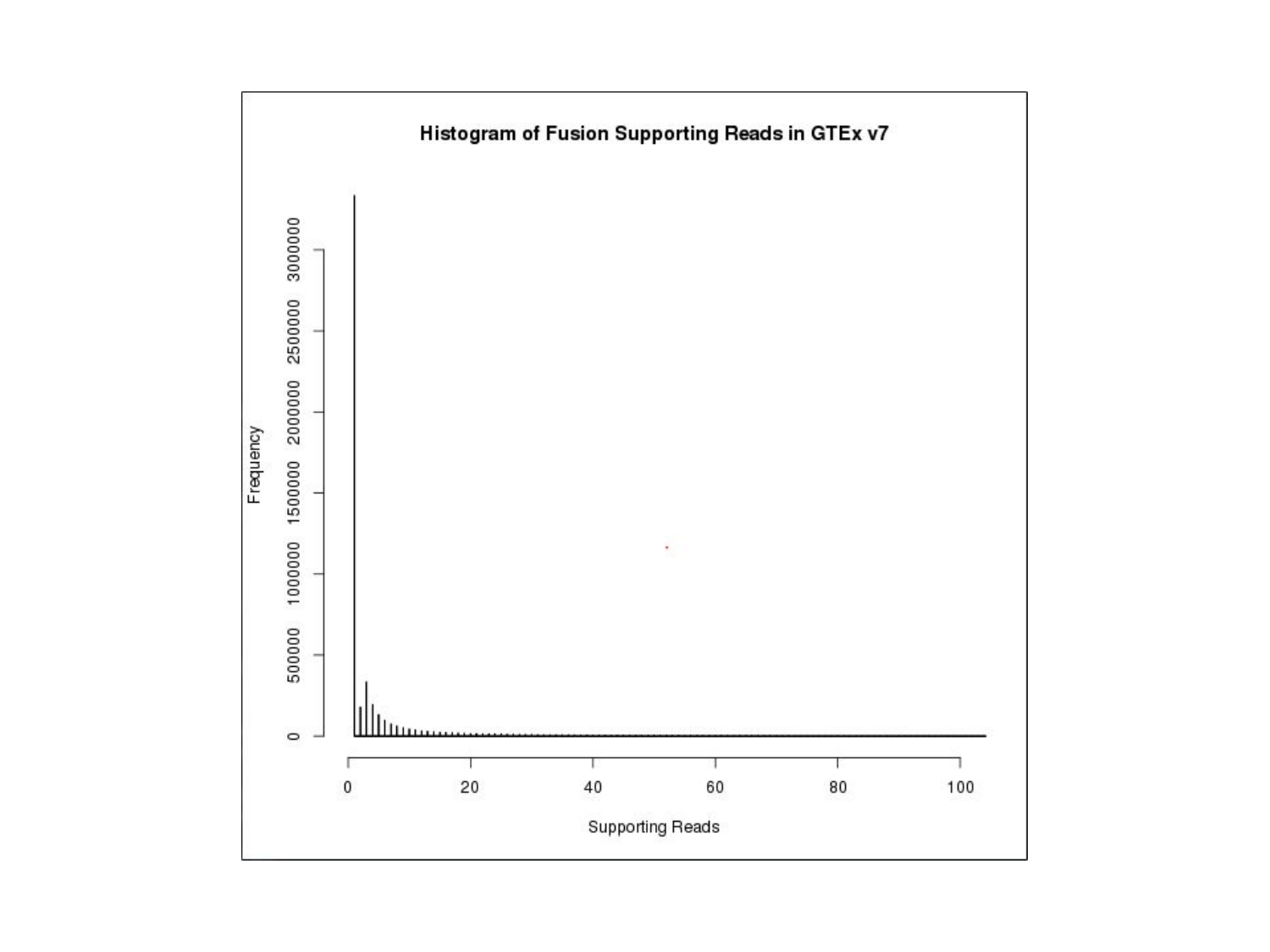

Supplement: S1 Fig — (PPTX) [file pone.0223337.s009.pptx]

## Slide 1
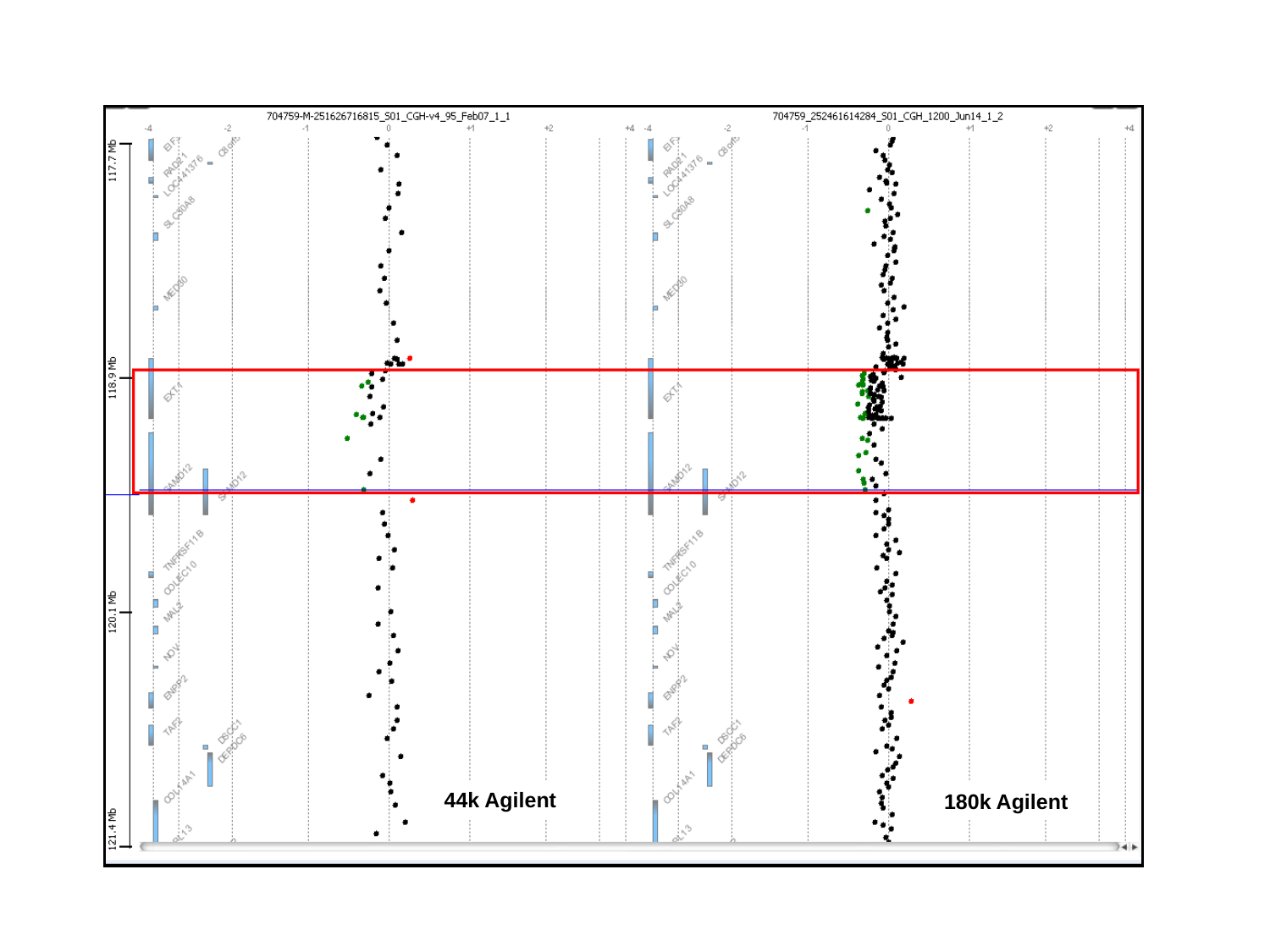

44k Agilent
180k Agilent

Supplement: S2 Fig — Despite initially negative clinical aCGH findings (Agilent 44k array), re-evaluation of sub calling threshold results suggested the presence of a mosaic deletion that was subsequently confirmed by increased density Agilent 180k array. (PPTX) [file pone.0223337.s010.pptx]

## Slide 1
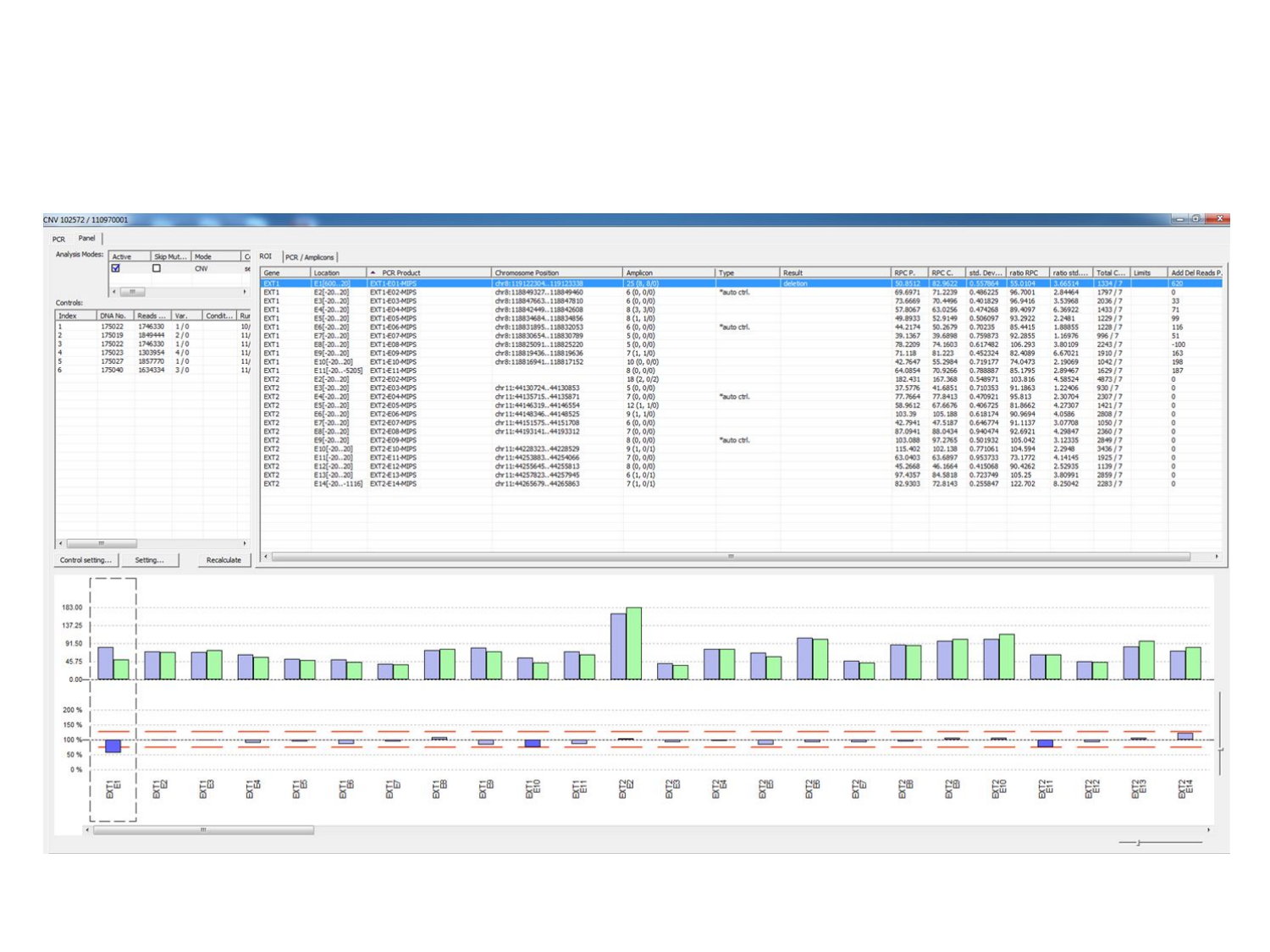

Supplement: S3 Fig — (PPTX) [file pone.0223337.s011.pptx]

## Slide 1
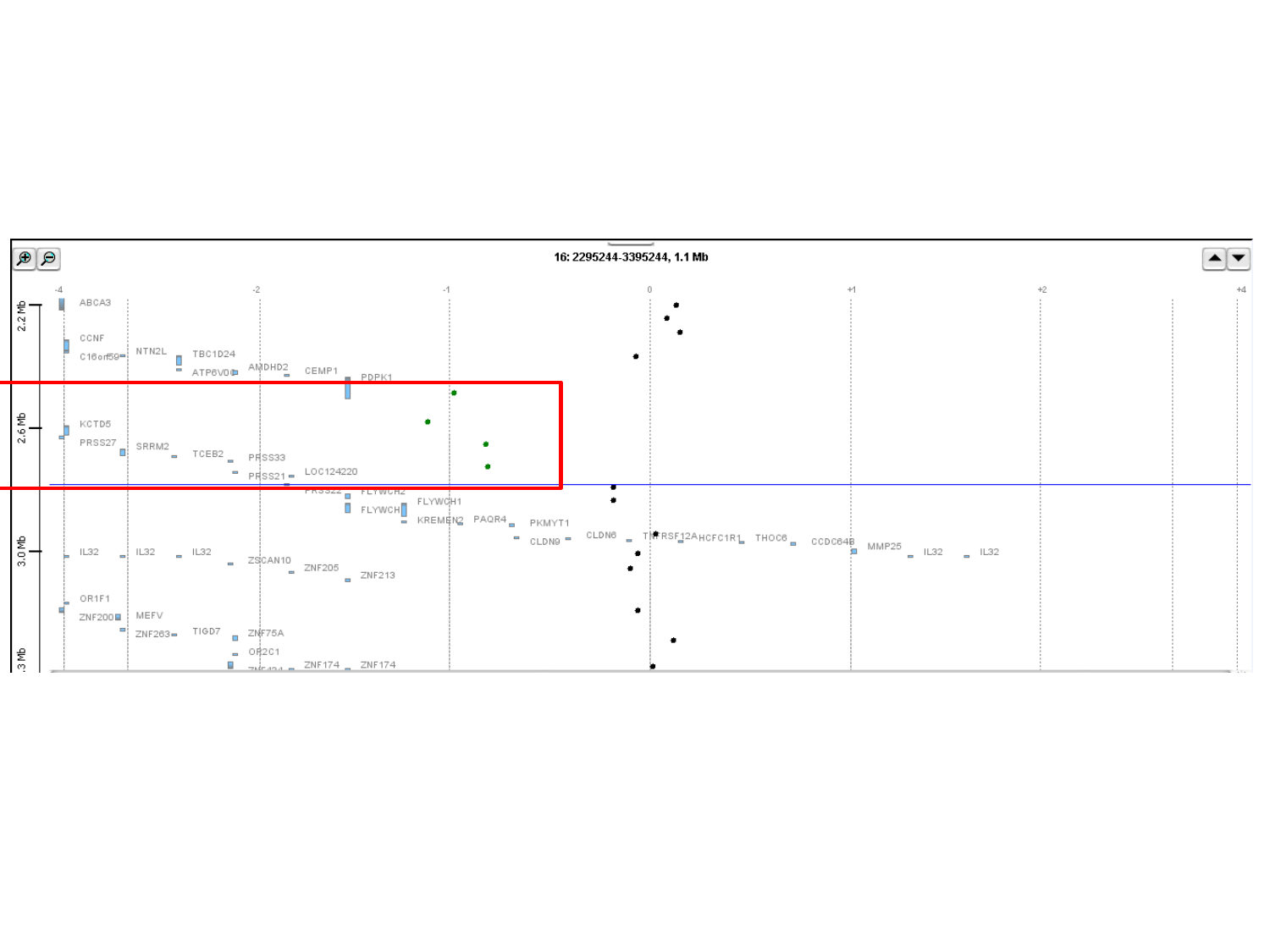

Supplement: S4 Fig — Reduced probe intensities and associated genes are demarcated by the red outline. PDPK1 and PRSS21 are seen at the boundaries. (PPTX) [file pone.0223337.s012.pptx]

## Slide 1
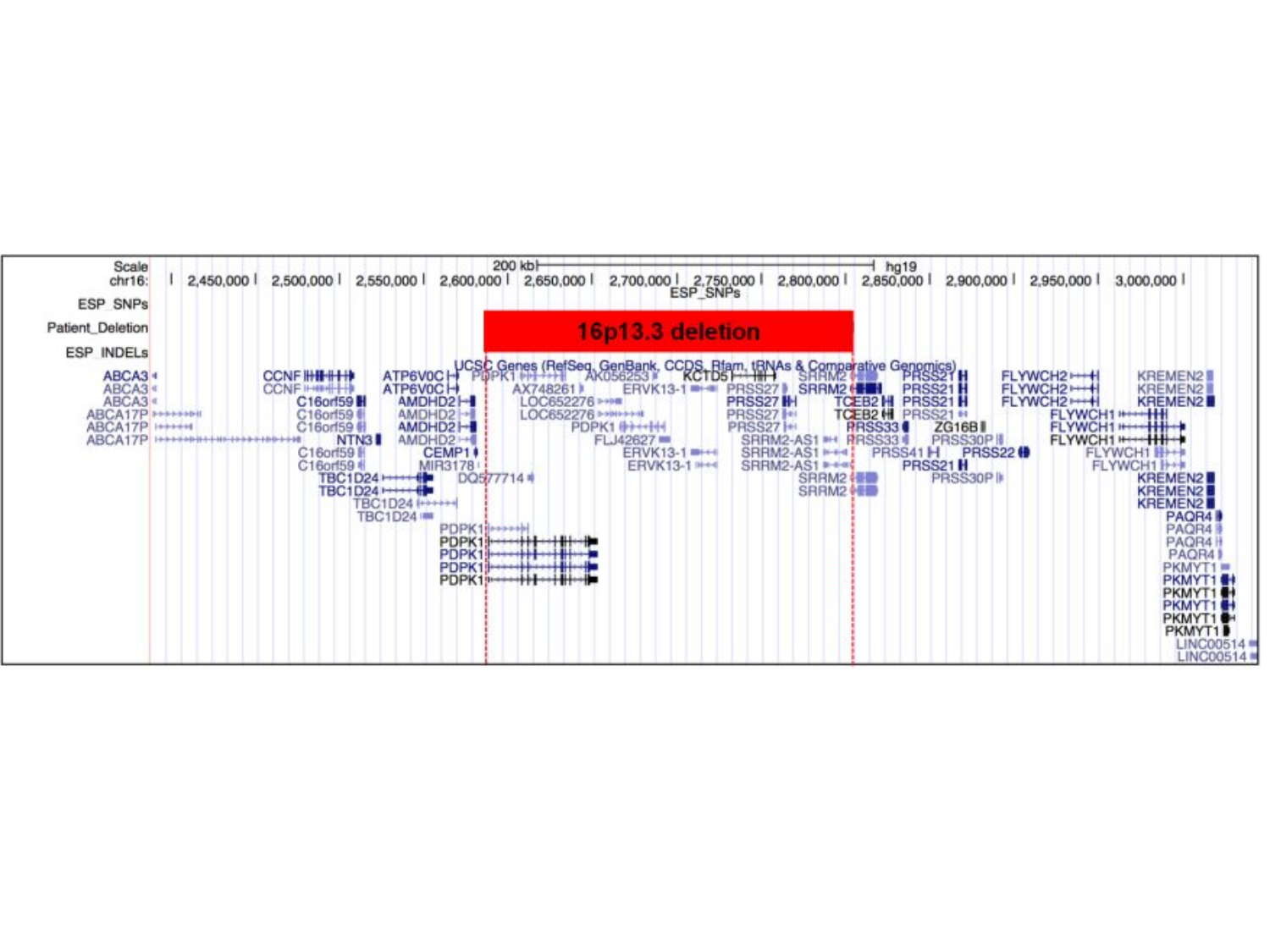

Supplement: S5 Fig — The deleted interval contained 10 genes with PDPK1 and PRSS21 lying at the 5’ and 3’ boundaries respectively. While a link to patient phenotype cannot be ruled out, the relevance of the deletion and fusion remain uncertain in the light of the co-occurring SAMD12-EXT1 fusion and DCX variant which were both classified as pathogenic. (PPTX) [file pone.0223337.s013.pptx]

## Slide 1
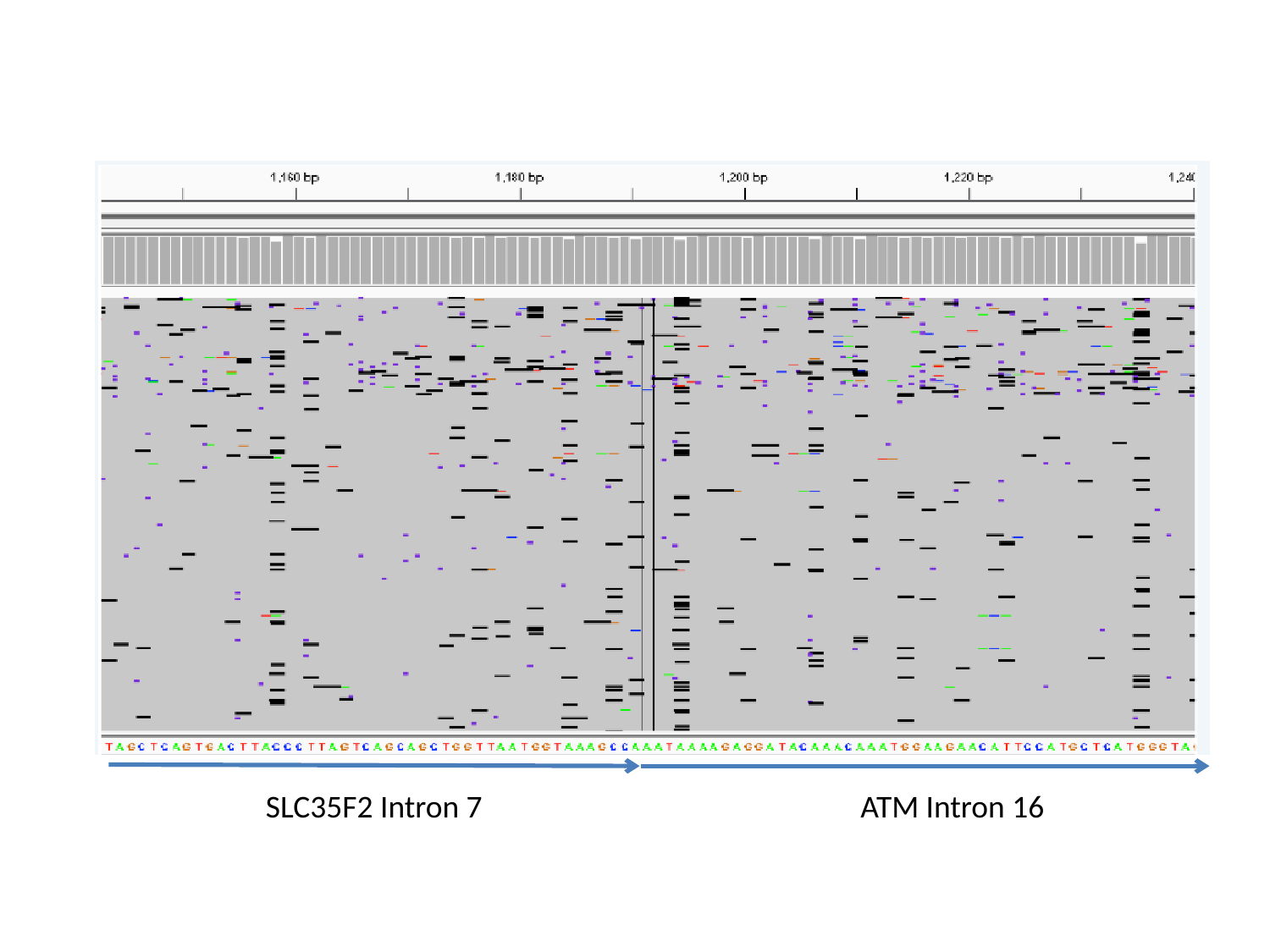

SLC35F2 Intron 7
ATM Intron 16

Supplement: S6 Fig — Reads are shown aligned to the fused sequence in window showing the breakpoint in SLC35F2 intron 7 and ATM intron 16. (PPTX) [file pone.0223337.s014.pptx]

## Slide 1
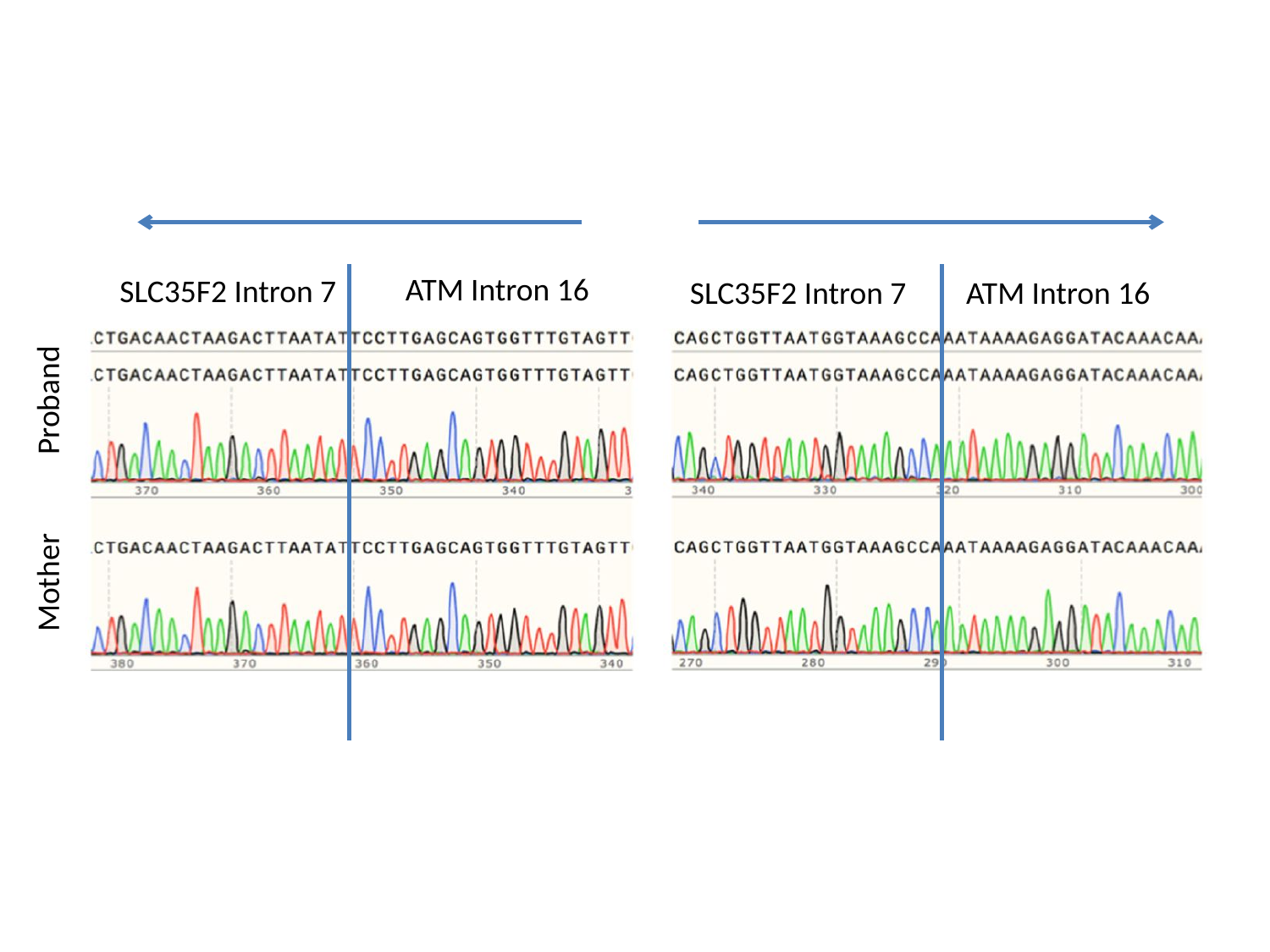

ATM Intron 16
SLC35F2 Intron 7
SLC35F2 Intron 7
ATM Intron 16
Mother Proband

Supplement: S7 Fig — (PPTX) [file pone.0223337.s015.pptx]
