# Supplementary material for: A tailored approach to fusion transcript identification increases diagnosis of rare inherited disease
Source: PLoS One. 2019 Oct 2;14(10):e0223337. doi: 10.1371/journal.pone.0223337 (PMC6774566; doi:10.1371/journal.pone.0223337)
Supplement: S1 File — (DOCX) [file pone.0223337.s016.docx]

| Fusion | Primer ID | Primer Sequence (5'-->3') | Product Size (bp) |
| --- | --- | --- | --- |
| SAMD12-EXT1 | SAMD12 Exon 1 Forward | CTGCCCATGCTGAAGGTATTAAAC | 139 |
|  | SAMD12 Exon 2 Reverse | TTAGCCGTCTCAGCTTCTGC |  |
|  | EXT1 Exon 2 Forward | TGATTATCGGGAAATGCTGCAC | 79 |
|  | EXT1 Exon 3 Reverse | CAGGAATCTGAAGGACCCAAGC |  |
|  | SAMD12 Exon 1 Forward | ATATTTAGGCGAAAGCGAGAGGAAG | 472 |
|  | EXT1 Exon 3 Reverse | CATCGCCTATGACGGCAGC |  |
| PDPK1-PRSS21 | PDPK1 Exon 7 Forward | TGAGGAAATGGAAGGATACGGACC | 335 |
|  | PDPK1 Exon 8 Reverse | ACAACAACCTCTTCTCATCTTCG |  |
|  | PRSS21 Exon 1 Forward | GACTGCTGGGTGACTGGCTG | 254 |
|  | PRSS21 Exon 2 Reverse | ACCCTGGTGCGTGTGTTGC |  |
|  | PDPK1 Exon 7 Forward/PRSS21 Exon 2 Reverse | Same as above | 356 |
| NARS2-TENM4 | NARS2 Exon 6 Forward | CAATGTTCCTGCTTTCTTAACTGTC | 136 |
|  | NARS2 Exon 7 Reverse | AAACTCTGCCAGGTGCCTC |  |
|  | TENM4 Exon 33 Forward | AGCTGGCTGCTCACCTTTG | 282 |
|  | TENM4 Exon 34 Reverse | AAAGACTGAGCCGCTGGATG |  |
|  | NARS2 Exon 6 Forward/TENM4 Exon 34 Reverse | Same as above | 204 |
| SON-FCRL3 | SON Exon 3 Forward | CCCAAGCCGCCGGAGAAGATCAAG | 170 |
|  | SON Exon 3 Reverse | TCAGACGTTTGGGTGATCTGCCTC |  |
|  | SON Pseudogene Forward | GCCGCCAGAGAAGATCAAAGTC | 492 |
|  | SON Pseudogene Reverse | AAATGGGTTTGGGTTCACTGAG |  |
|  | FCRL3 Exon 1 Forward | ACATTACTCCTGAGAGACTGC | 209 |
|  | FCRL3 Exon 2 Reverse | AGCAGCCACAGAAGCATG |  |
|  | SON-FCRL3 Fusion Forward | TCAGACGTTTGGGTGATCTGCCTC | 224 |
|  | SON-FCRL3 Fusion Reverse | AGCAGCCACAGAAGCATG |  |
|  | SON-FCRL3 Fusion Nested Forward | TCGTTCAGAAGACCTGGATC | 174 |
|  | SON-FCRL3 Fusion Nested Reverse | GGGCAGAGGGTTGGGAAAG |  |
|  | SON Pseudo-FCRL3 Fusion Forward | AAATGGGTTTGGGTTCACTGAG | 527 |
|  | SON Pseudo-FCRL3 Fusion Reverse | AGCAGCCACAGAAGCATG |  |
|  | SON Pseudo-FCRL3 Fusion Nested Forward | GGAGGTTCTTCTTCCTCTTCATC | 455 |
|  | SON Pseudo-FCRL3 Fusion Nested Reverse | GGGCAGAGGGTTGGGAAAG |  |
| ATM-SLC35F2 (and SLC35F2-ATM) | Cousin *et al 2018* |  |  |
| SLC30A6-SPAST | SLC30A6 Exon 5 Forward | GTTTAATAAGTTACTGGGTAACATTGAGG | 115 |
|  | SLC30A6 Exon 6 Reverse | GCTCCCAACTGTGCCAAGAC |  |
|  | SPAST Exon 5 Forward | GCCTTTCAGGCCACCATAG | 225 |
|  | SPAST Exon 6 Reverse | CCACAATTTCATTCATTATAAGGTTAGC |  |
|  | SLC30A6 Exon 5 Forward/SPAST Exon 6 Reverse | Same as above | 185 |
|  | SLC30A6 Exon 5 Nested Forward | GTAACATTGAGGAAACCTAGCCCTG | 139 |
|  | SPAST Exon 6 Nested Reverse | AGGTTGCTGTCCACATTCC |  |
| CACNB4-STAM2 | CACNB4 Exon 2 Forward | ACCACAACCCGGAGGAGCAG | 178 |
|  | CACNB4 Exon 3 Reverse | GCTGGATAGCTGCTTGCTG |  |
|  | STAM2 Exon 1 Forward | GGGATCGAAGGGTGGACTC | 285 |
|  | STAM2 Exon 2 Reverse | GTCACATATGTCCATAATAAGACTCC |  |
|  | CACNB4 Exon 2 Forward/STAM2 Exon 2 Reverse | Same as above | 134 |
|  | CACNB4 Exon 2 Nested Forward | AAAGATCCGATGGCAGCAC | 81 |
|  | STAM2 Exon 2 Nested Reverse | TCTTCTGTAGTGTTGTACTCATTCG |  |
| RPL17/C18orf32-DYM | RPL17/C18orf32 Exon 6 Forward | TATCGTCATTCCAGTTCTGCTC | 292 |
|  | RPL17/C18orf32 Exon 7 Reverse | TCAGAGACAATTACAAGGAAGATGC |  |
|  | DYM Exon 1 Forward | GGATCTGTACCCGCTGAG | 325 |
|  | DYM Exon 2 Reverse | GAGAGAAGCTGATTCCAGAACG |  |
|  | RPL17/C18orf32 Exon 6 Forward/DYM Exon 2 Reverse | Same as above | 314 |
|  | RPL17/C18orf32 Exon 6 Nested Forward | TGGCCTAAGAAAGCAATACAAG | 200 |
|  | DYM Exon 2 Nested Reverse | ATTCTCAGAGATAGATTCCGTGC |  |
| TET3-DGUOK | TET3 Exon 2 Forward | TGGAAATAAAGGCTGGTGAAGGAG | 276 |
|  | TET3 Exon 3 Reverse | GGCGAGCAGTATGAGCTGG |  |
|  | DGUOK Exon 2 Forward | TGGGAAAGTCCACGTTTGTGAAG | 222 |
|  | DGUOK Exon 3 Reverse | GCTGTACTTTCAGGCGGCTC |  |
|  | TET3 Exon 2 Forward/DGUOK Exon 3 Reverse | Same as above | 168 |
|  | TET3-DGUOK Nested | GGGCCGTGGGGACAAGG | 87 |
|  | TET3-DGUOK Nested | TGCTGGCTCCCGGTACATC |  |
| ARL5A-NEB | ARL5A Exon 6 Forward | TGTGCCAAGGACTTGAATGGATG | 453 |
|  | ARL5A Exon 7 Reverse | TCTGAACTGAGAGCTTCATCC |  |
|  | NEB Exon 1 Forward | GGAGAGTTGGGAGAGGCTTTG | 114 |
|  | NEB Exon 2 Reverse | CTTCCCGAACACCATTGGCTTATAC |  |
|  | ARL5A Exon 6 Forward/NEB Exon 2 Reverse | Same as above | 378 |
|  | ARL5A Exon 6 Nested Forward | TGCTGGACTTTACCTGAAAGCTG | 260 |
|  | NEB Exon 2 Nested Reverse | TTTCTTTCGTTTCTGTAGCTCTCG |  |
