# Supplementary material for: A tailored approach to fusion transcript identification increases diagnosis of rare inherited disease
Source: PLoS One. 2019 Oct 2;14(10):e0223337. doi: 10.1371/journal.pone.0223337 (PMC6774566; doi:10.1371/journal.pone.0223337)
Supplement: S2 File — (DOCX) [file pone.0223337.s017.docx]

| **Primer Name** | **Sequence (5'-->3')** | **Amplicon (bp)** |
| --- | --- | --- |
| SON anchored Forward | CTTGATCGCCTTAATCTGACTG | 65 |
| FCRL3 anchored Reverse | TATCTCCAAGAAGGAGGGCAG |  |
|  |  |  |
| C18or32 partially anchored Forward | GCCTAAGAAAGCAATACAAGAATCC | 90 |
| DYM anchored reverse | TTGTCCTTAAACCTGCATTTCC |  |
|  |  |  |
| CACNB4 anchored forward | GATCCGATGGCAGCACCAC | 74 |
| STAM2 anchored reverse | CTGTAGTGTTGTACTCATTCGTGG |  |
|  |  |  |
| SLC30A6 anchored forward | ACCTAGCCCTGTCTATTCATTTG | 125 |
| SPAST reverse (not anchored) | AGGTTGCTGTCCACATTCC |  |
|  |  |  |
| NARS2 anchored forward | ACTGTCTCAGGACAACTTCATC | 75 |
| TENM4 partially anchored reverse | GCTGCTTCTGTACTTCACAC |  |
|  |  |  |
| TET3 anchored forward | GACAAGGAGCGGCTGTCAAG | 65 |
| DGUOK anchored reverse | GTACATCATATCCAGCAAGTTTCC |  |
|  |  |  |
| ARL5A anchored forward | ATCCTCTCAGGACATTGTGTAGC | 58 |
| NEB anchored forward | TTCTTTCGTTTCTGTAGCTCTCG |  |
|  |  |  |
| SAMD12 anchored forward | GAAAGGAACTCCCAAGCGAC | 83 |
| EXT1 partially anchored reverse | GAAAGTGGCATTGTGCAGC |  |
|  |  |  |
| PDPK1 anchored forward | CGGCTATGTCGGAAGACG | 82 |
| PRSS21 anchored reverse | CTTCCTGGCGAGCAGAC |  |
|  |  |  |
| ATM forward (NOT anchored) | TGCATCTGGCTTTTTCCTG | 114 |
| SLC35F2 anchored reverse | ACCATGATGACAGTGAAGGAC |  |
|  |  |  |
| SLC35F2 anchored forward | TTGTTGGACTCTTTCTGTTTGG | 77 |
| ATM anchored reverse | CATTGATTCTACTTCTCCACGGTC |  |
